# PPFIA1 drives active α5β1 integrin recycling and controls fibronectin fibrillogenesis and vascular morphogenesis

**DOI:** 10.1038/ncomms13546

**Published:** 2016-11-23

**Authors:** Giulia Mana, Fabiana Clapero, Emiliano Panieri, Valentina Panero, Ralph T. Böttcher, Hui-Yuan Tseng, Federico Saltarin, Elena Astanina, Katarzyna I. Wolanska, Mark R. Morgan, Martin J. Humphries, Massimo M. Santoro, Guido Serini, Donatella Valdembri

**Affiliations:** 1 Department of Oncology, University of Torino School of Medicine, Candiolo, Torino 10060, Italy; 2 Laboratory of Cell Adhesion Dynamics, Candiolo Cancer Institute—Fondazione del Piemonte per l'Oncologia (FPO), Istituto di Ricovero e Cura a Carattere Scientifico (IRCCS), Candiolo, Torino 10060, Italy; 3 Department of Molecular Biotechnology and Health Sciences, Molecular Biotechnology Center, University of Torino, 10126 Torino, Italy; 4 Department of Molecular Medicine, Max Planck Institute of Biochemistry, Martinsried 82152, Germany; 5 Laboratory of Vascular Oncology, Candiolo Cancer Institute—Fondazione del Piemonte per l'Oncologia (FPO), Istituto di Ricovero e Cura a Carattere Scientifico (IRCCS), Candiolo, Torino 10060, Italy; 6 Department of Cellular and Molecular Physiology, Institute of Translational Medicine, University of Liverpool, Liverpool L69 3BX, UK; 7 Wellcome Trust Centre for Cell-Matrix Research, Faculty of Biology, Medicine & Health, University of Manchester, Manchester M13 9PT, UK; 8 Laboratory of Endothelial Molecular Biology, Vesalius Research Center, VIB, Leuven B-3000, Belgium

## Abstract

Basolateral polymerization of cellular fibronectin (FN) into a meshwork drives endothelial cell (EC) polarity and vascular remodelling. However, mechanisms coordinating α5β1 integrin-mediated extracellular FN endocytosis and exocytosis of newly synthesized FN remain elusive. Here we show that, on Rab21-elicited internalization, FN-bound/active α5β1 is recycled to the EC surface. We identify a pathway, comprising the regulators of post-Golgi carrier formation PI4KB and AP-1A, the small GTPase Rab11B, the surface tyrosine phosphatase receptor PTPRF and its adaptor PPFIA1, which we propose acts as a funnel combining FN secretion and recycling of active α5β1 integrin from the *trans-*Golgi network (TGN) to the EC surface, thus allowing FN fibrillogenesis. In this framework, PPFIA1 interacts with active α5β1 integrin and localizes close to EC adhesions where post-Golgi carriers are targeted. We show that PPFIA1 is required for FN polymerization-dependent vascular morphogenesis, both *in vitro* and in the developing zebrafish embryo.

## Abstract (web summary)

During vascular development, fibronectin (FN) is polymerized at the basolateral side of endothelial cells. Here Mana *et al*. propose a model where PPFIA1 drives recycling of the FN receptor, a5β1 integrin, to the cell surface and enables polar secretion and fibrillogenesis of newly synthesized FN.

## Graphical abstract

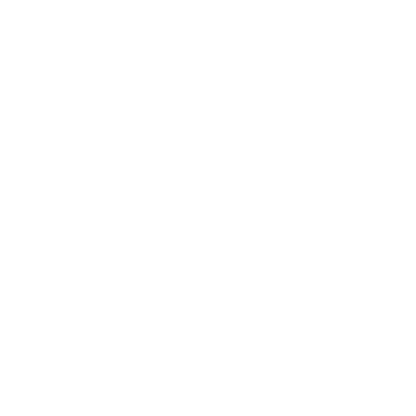

Essentially all morphogenetic events of multicellular organisms, including angiogenesis^1^, require a highly dynamic adhesion to extracellular matrix (ECM) proteins that is mediated primarily by integrins^2^. During vascular development, the interaction between fibronectin (FN) and its major receptor α5β1 integrin induces apico-basal polarity of endothelial cells (ECs) and the formation of the single lumen of blood vessels^3^,^4^. Integrin-mediated ECM signalling controls the spatial orientation of the apico-basal axis not only in ECs^5^, but also in several other polarized cell types^6^. Cells secrete FN as a soluble dimer that is assembled by integrins into a fibrillar network. The latter represents the bioactive form of FN and is capable of inducing mechanical and chemical signals that are essential for EC tubulogenesis^7^ and *in vivo* angiogenesis^1^,^8^. Although the fibrillar FN network is cross-linked and associated with other ECM proteins^8^, it is continuously turned over and remodelled due to matrix metalloproteinase-dependent cleavage^9^ and integrin-mediated endocytosis of freed FN proteolytic fragments^10^,^11^,^12^,^13^. Indeed, a complex machinery composed of the transmembrane glycoprotein neuropilin 1 (Nrp1), its cytosolic interactor GAIP interacting protein C terminus, member 1 (GIPC1) and the associated motor myosin VI (MYO6) selectively controls the endocytosis of FN fragments bound to active α5β1 integrins from the surface of ECs^11^,^14^. The importance of this process is highlighted by the severe impairment of FN fibrillogenesis that occurs in ECs on disruption of Nrp1 signaling^14^. Since internalized active α5β1 integrins recycle back to the cell surface^15^,^16^, it has been proposed that they may also accomplish other tasks in addition to the endocytosis of FN fragments, such as the binding of newly synthesized FN in the exocytosis pathway, resulting in its deposition and polymerization at the basolateral surface of ECs to replenish degraded FN fibrils^9^,^10^,^14^. Despite the attractiveness of this hypothesis and the fact that the turnover of the fibrillar FN network is essential for physiologic as well as pathologic angiogenesis, it is unknown where in the cell α5β1 integrins may be loaded with newly synthesized FN molecules and how the exocytosis of the FN-α5β1 integrin complex could be orchestrated at the molecular level.

In polarized cells, the asymmetric distribution of transmembrane and secreted soluble proteins, for example, the selective basolateral deposition of FN fibrils in ECs^17^,^18^,^19^, relies on the unique sorting properties of the TGN along the biosynthetic routes^20^,^21^. Specific apical and basal endosomal compartments are thought to control the local recycling of endocytosed receptors^22^. However, recent studies suggest that internalized integrins may also reach the TGN before being recycled back to the plasma membrane^23^,^24^,^25^,^26^. Liprins α and β (ref. 27) are widely expressed and related adaptor proteins that, by interacting with the protein tyrosine phosphatase receptor type f polypeptide (PTPRF, also known as leukocyte common antigen related—LAR protein)^28^, were originally found to control synapse formation and function^29^,^30^. Notably, in addition to cooperating with liprin β in the control of synapse size^30^, PTPRF interacting protein α1 (PPFIA1), otherwise known as liprin-α1 (ref. 27), also coordinates presynaptic endo-exocytic traffic and drives the docking of neurotransmitter-containing vesicles with the plasma membrane^29^,^31^,^32^. Here, we identify PPFIA1 as a key component of the molecular machinery that in ECs physically binds and controls the recycling of endocytosed active α5β1 integrin to the cell surface and the basolaterally-polarized fibrillogenesis of newly synthesized FN.

## Results

### PPFIA1 binds to and controls active α5β1 integrin recycling

**Figure 1:**
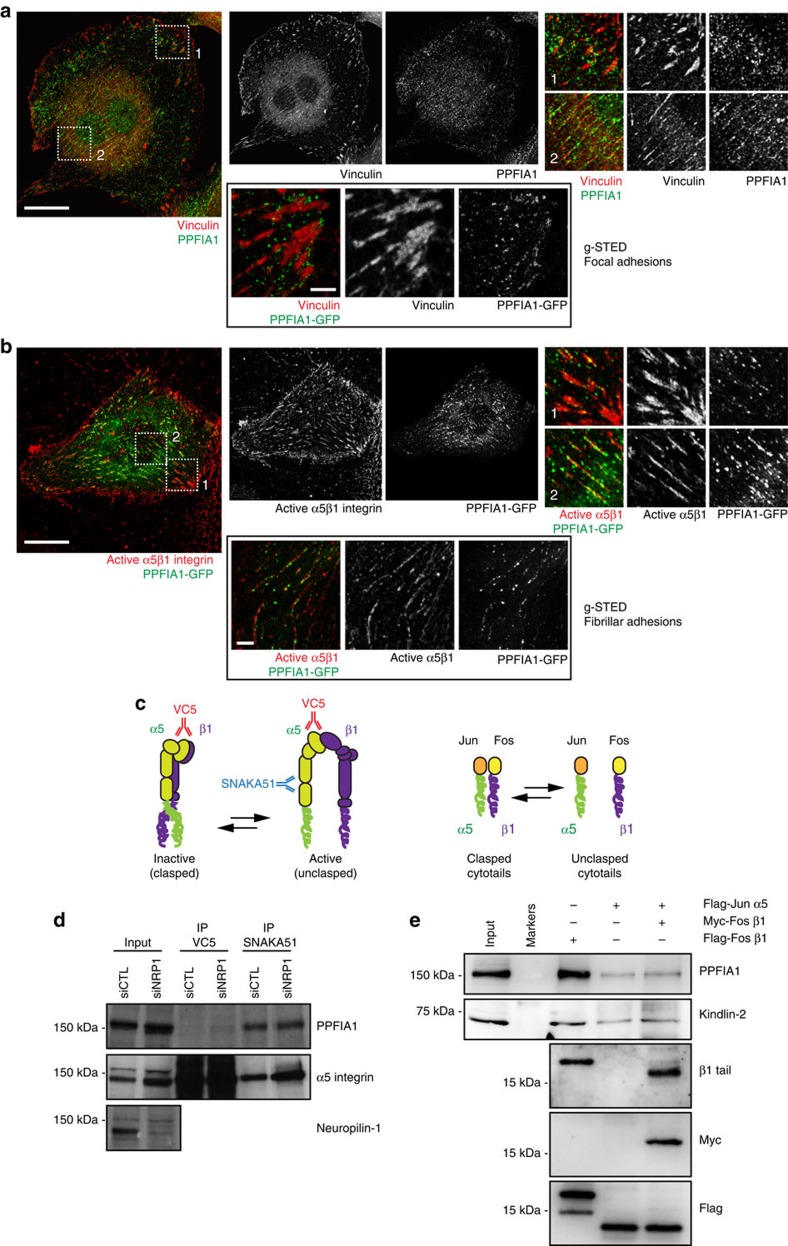
PPFIA1 localizes in proximity of EC fibrillar adhesions and selectively interacts with active α5β1 integrin cytodomain. (**a**,**b**) Conventional or g-STED (boxed) confocal microscopy analyses of endogenous PPFIA1 (green) or PPFIA1-GFP (green) and vinculin (red) or SNAKA51^+^ active α5β1 integrin (red) in ECs indicates that PPFIA1 localizes close to both focal and fibrillar adhesions. Right-sided panels **1** (focal adhesions) and **2** (fibrillar adhesions) are magnifications of boxed areas in left-sided panels. (**c**) Representation of inactive and active α5β1 integrin displaying clasped or unclasped cytotails, respectively. Epitopes recognized by VC5 and SNAKA51 mAbs are shown. VC5 binds to a β propeller domain epitope of the α5 subunit of both inactive and active α5β1 integrin, while SNAKA51 binds to the calf domains of the α5 subunit only when α5β1 integrin is active. Jun or Fos N-terminally-tagged recombinant integrin cytotails were exploited to mimic α5β1 integrin activation state. Jun-Fos dimerization and clasping of integrin cytotails, mimicked inactive α5β1 integrin. (**d**) Immunoprecipitation from EC lysates of total (that is, inactive and active) or active α5β1 integrin by VC5 and SNAKA51 mAbs, respectively, followed by western blotting with anti-PPFIA1 or anti-α5 integrin subunit or anti-Nrp1 antibodies. In ECs, PPFIA1 associates with active, but not inactive α5β1 integrin in both siCTL and siNRP1 ECs. Total EC lysates (input) were employed for control purposes. (**e**) Pull-down from mouse fibroblast cell lysates of PPFIA1 on isolated or dimerized α5 or β1 integrin cytotails. Kindlin-2 was pulled down for control purposes. Both PPFIA1 and kindlin-2 are pulled down on isolated Myc-Fos β1, but not Flag-Jun α5 integrin subunit cytotail. Furthermore, both interactions are severely impaired by forced Jun-Fos-mediated dimerization of α5 and β1 integrin subunit cytotails. α5 or β1 integrin cytotails are revealed by means of Western Blot with the respective anti-tag antibodies (Flag and/or Myc) or anti-β1 integrin subunit cytotail (for loading control purposes). Scale bar, 20 μm (**a**,**b**) and 2 μm (boxed g-STED panels).

We have previously identified Nrp1 and additional adaptor and motor proteins as key components of a signalling complex that selectively promotes endocytosis of active α5β1 integrin and the polymerization of FN into a basolateral fibrillar network by ECs^11^,^14^. While these findings highlight endocytic trafficking events involving active α5β1 integrins^11^,^14^ for the turnover of FN fibrils^9^,^10^, it is unclear whether the endocytosed active α5β1 integrins only remove fibrillar FN or whether they also replenish it. To address this question, we determined the subcellular localization of the ubiquitously expressed adaptor protein PPFIA1 that has been shown to couple endocytosis and exocytosis in presynaptic nerve termini^32^. Standard confocal microscopy analysis revealed that, as previously reported^33^, fluorescently immunostained PPFIA1 localized around mature peripheral focal adhesions of ECs (Fig. 1a, magnified panel 1). Furthermore, PPFIA1 also localized to the vicinity of centrally located vinculin-containing fibrillar adhesions (Fig. 1a, magnified panel 2), where active α5β1 integrins, as recognized by SNAKA51 monoclonal antibody (mAb)^34^,^35^, predominantly localized^14^. Similarly, when transfected in ECs, PPFIA1-GFP concentrated in close proximity to focal (Fig. 1b, magnified panel 1) and fibrillar (Fig. 1b, magnified panel 2) adhesions. These findings were further substantiated by super-resolved timed-gated stimulated emission depletion (g-STED) confocal microscopy analyses of PPFIA1-GFP transfected and anti-vinculin-stained or anti-active α5β1 integrin-stained ECs (Fig. 1a,b, g*-STED*).

To test whether PPFIA1 represents a novel regulator of active α5β1 integrin recycling, we first assessed whether PPFIA1 binds α5β1 integrins, and if NRP1 mediates this interaction. Lysates from ECs oligofected with either control siRNA (siCTL) or a pool of four siRNA targeting NRP1 (siNRP1) were immunoprecipitated with mAbs recognizing either total (that is, active and inactive, clone VC5) or active (clone SNAKA51) α5β1 integrins^36^ (Fig. 1c, left panel). PPFIA1 co-immunoprecipitated with SNAKA51^+^/active α5β1 integrins, but NRP1 did not mediate the interaction (Fig. 1d). Indeed, quantitative confocal analysis of relative surface fluorescence of individual ECs showed that SNAKA51^+^ active α5 integrin subunits account for the 10% of VC5^+^ or AB1928^+^ total α5 integrin subunits (Supplementary Fig. 1).

To corroborate the interaction between active α5β1 integrin and PPFIA1, we performed pull-down assays from cell lysates using recombinantly expressed and purified monomeric or dimeric α5 or β1 integrin cytotails. Complementary DNAs encoding the cytoplasmic domain of either α5 or β1 integrin subunit were fused at their N-terminus with Jun and Fos peptides, respectively (Fig. 1c, right panel). To mimic the unclasped and active or the clasped and inactive α5β1 integrin conformation, recombinant α5 and β1 cytotails were employed alone or in combination to force their dimerization and clasping via the high affinity Jun-Fos interaction (Fig. 1c, right panel). The well-known β1 integrin cytosolic interactor kindlin 2 (ref. 37) was used as a positive control. Both PPFIA1 and kindlin 2 were pulled down by isolated Fos β1, but not Jun α5 integrin subunit cytotails. Furthermore, the interactions of both proteins were significantly impaired by forced Jun-Fos-mediated dimerization of α5 and β1 integrin subunit cytotails (Fig. 1e).

**Figure 2:**
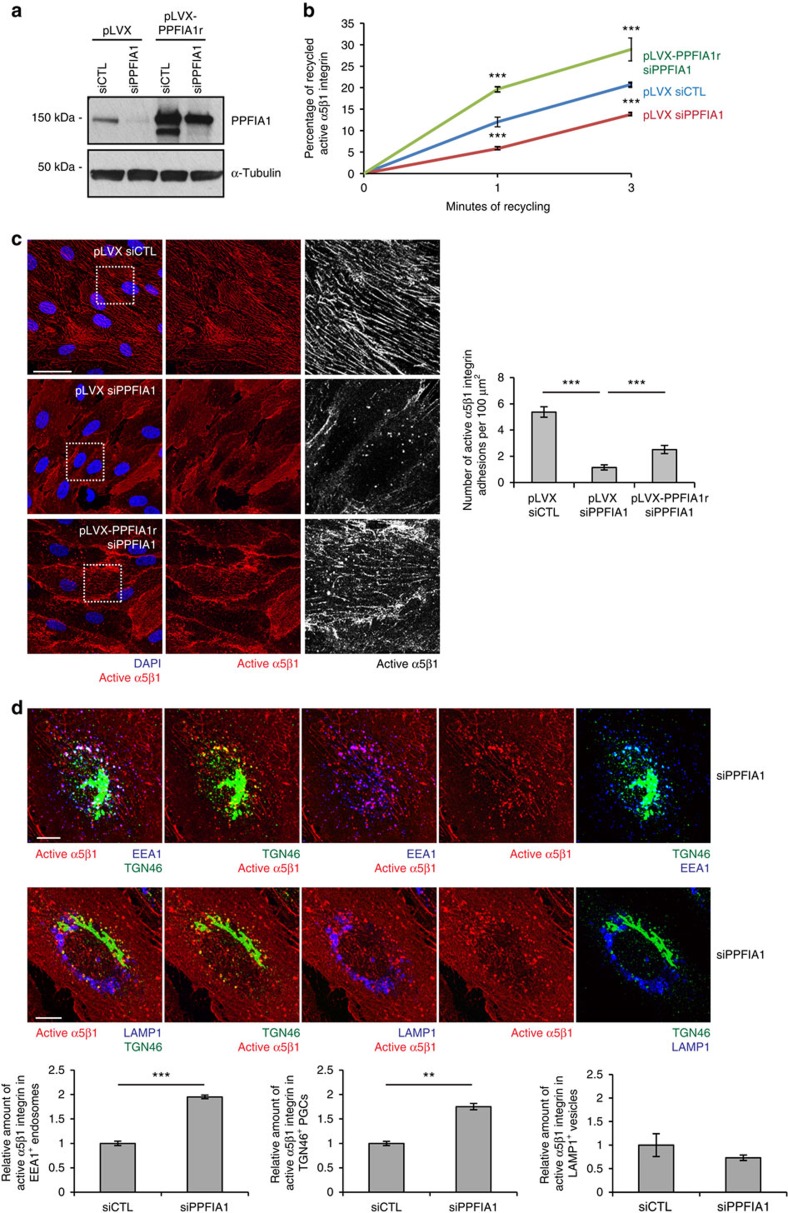
PPFIA1 drives the recycling of endocytosed active α5β1 integrin. (**a**) Western blot analysis of EC lysates, transduced with pLVX lentivirus carrying or not the silencing-resistant PPFIA1 construct (PPFIA1r) and then control (siCTL) or PPFIA1 (siPPFIA1) silenced. (**b**) Time-course analysis of the relative amounts of recycled active α5β1 integrin in pLVX siCTL ECs versus pLVX siPPFIA1 ECs or siPPFIA1 ECs rescued with pLVX-PPFIA1r, evaluated by integrin recycling assay and capture ELISA assay. PPFIA1 silencing significantly impairs the recycling of active α5β1 integrin. Values are mean±s.e.m., *n*=3 independent experiments (two technical replicates for each experiment). (**c**) Confocal microscopy analysis of anti-active α5β1 integrin mAb SNAKA51 localization (red) in living confluent ECs following 20 min of incubation. SNAKA51^+^ active α5β1 integrin localizes in fibrillar adhesion in pLVX siCTL, but not in pLVX siPPFIA1 ECs in which it accumulates instead in perinuclear punctae. PLVX-mediated PPFIA1r overexpression restores the fibrillar adhesion localization of SNAKA51 in siPPFIA1 ECs. The number of active α5β1 integrin-containing adhesions per 100 μm^2^ of cell area was quantified in pLVX siCTL, pLVX siPPFIA1 or pLVX-PPFIA1r+siPPFIA1 ECs. Data are mean values±s.e.m., *n*=20 cells per condition pooled from two independent experiments. (**d**) Fluorescent confocal microscopy analysis of EC perinuclear punctae in which SNAKA51^+^ active α5β1 integrin accumulates on PPFIA1 silencing. In siPPFIA1 ECs, SNAKA51^+^ active α5β1 integrin is enriched in a perinuclear vesicular compartment labelled by both TGN (TGN46) and early endosome (EEA1), but not late endosome (LAMP1) markers. Relative amount of fluorescence intensity of SNAKA51^+^ active α5β1 integrin, respectively on EEA1^+^ endosomes or on TGN46^+^ PGCs, was evaluated. Data are mean value±s.e.m., *n*=70 cells per condition pooled from two independent experiments **P<0.01; ***P<0.001; Student's *t*-test. Scale bar, 50 μm (**c**), 10 μm (**d**).

In ECs, PPFIA1 localizes in close proximity to fibrillar adhesions and interacts with the β1 cytotail of active α5β1 integrins. Hence, we sought to investigate whether, in line with the role it plays in neuronal synapses^31^,^32^, PPFIA1 participates in the recycling of endocytosed active α5β1 integrins. By means of biochemical assays^38^, we first analyzed the extent of total or active α5β1 integrin recycling at different time points on internalization. In accordance with previous quantitative fluorescence confocal microscopy analyses^39^, we found that total α5β1 integrin rapidly recycled at early (5 min), but not late (10–15 min) time points after internalization (Supplementary Fig. 2a). By contrast, active α5β1 integrin was more efficiently recycled back to the EC surface at late (15 min) rather than early (5 min) time points after internalization (Supplementary Fig. 2b). Next, we studied the effects of PPFIA1 silencing (siPPFIA1, Fig. 2a) on active α5β1 integrin recycling in ECs. pLVX lentivirus-driven overexpression of an siRNA-resistant PPFIA1 (pLVX-PPFIA1r) was employed to rescue PPFIA1 expression levels (Fig. 2a). Notably, when compared with control transduced (pLVX) and silenced (siCTL) ECs, PPFIA1 knockdown (pLVX siPPFIA1) significantly reduced the amount of recycled active α5β1 integrin (Fig. 2b). Importantly, pLVX-PPFIA1r restored the defective recycling phenotype of PPFIA1-silenced (pLVX-PPFIA1r siPPFIA1) ECs (Fig. 2b).

To determine whether impaired active α5β1 integrin recycling may correlate with its altered subcellular localization, we incubated SNAKA51 mAb for 20 min at 37 °C on confluent living pLVX siCTL or pLVX siPPFIA1 ECs that were then processed for immunofluorescence and analyzed by confocal microscopy. In comparison with pLVX siCTL ECs, where SNAKA51 mainly localized in α5β1-containing fibrillar adhesions, in pLVX siPPFIA1 ECs SNAKA51^+^/active α5β1 integrins accumulated in perinuclear punctae (Fig. 2c). Reintroduction of PPFIA1r rescued the fibrillar distribution of active α5β1 integrin in PPFIA1-silenced (pLVX-PPFIA1r siPPFIA1) ECs (Fig. 2c). Furthermore, fluorescence confocal microscopy analyses of perinuclear punctae revealed that, in siPPFIA1 ECs, internalized active α5β1 integrin accumulated in TGN46^+^ vesicles, which correspond to bona fide post-Golgi carriers (PGCs) and, in accordance with previous findings^40^,^41^, are often positive for the early endosome antigen 1 (EEA1) (Fig. 2d, upper panels). The negligible fraction of internalized SNAKA51^+^/active α5β1 integrins that colocalize with the late endosome marker lysosomal-associated membrane protein 1 (LAMP1) was not affected by PPFIA1 silencing (Fig. 2d, lower panels).

**Figure 3:**
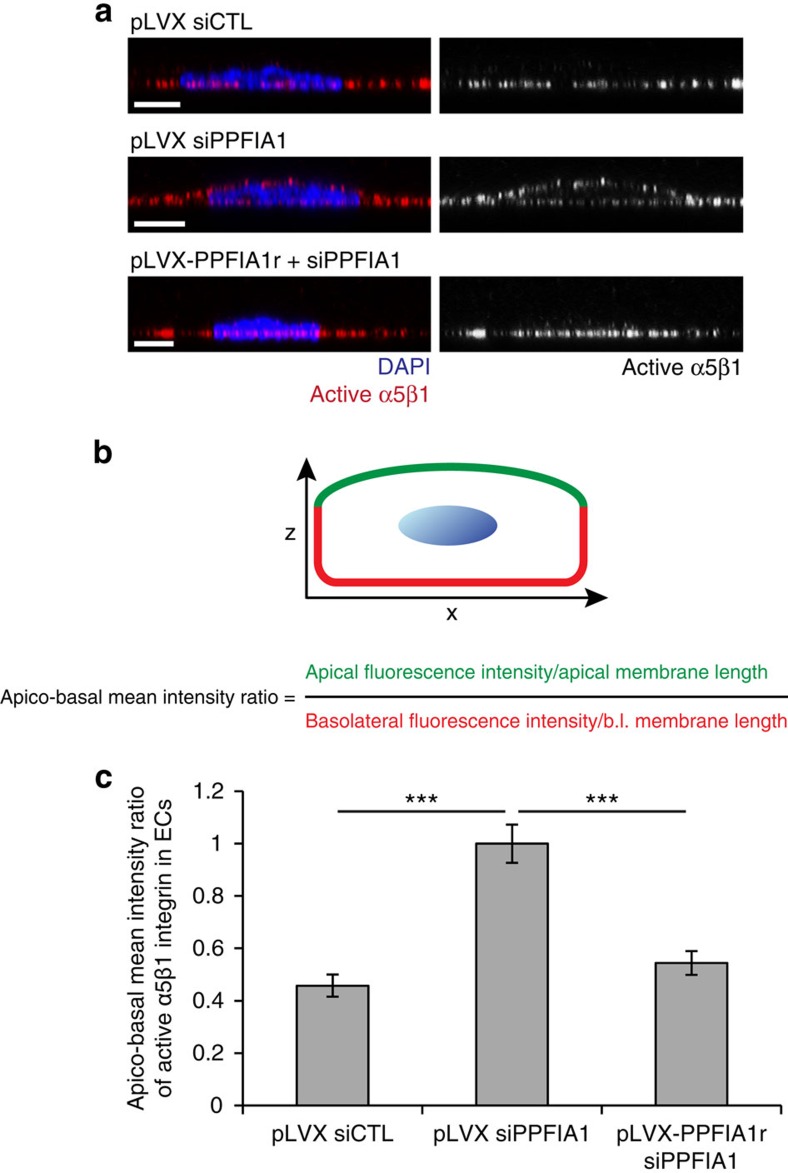
PPFIA1 drives basolateral localization of active α5β1 integrin. (**a**) Confocal xz sectioning microscopy analysis of anti-active α5β1 integrin cell surface localization (red) following 20 min of incubation with SNAKA51 mAb on living confluent ECs. (**b**) Schematic representation of the quantification of apico-basal mean intensity ratio of SNAKA51^+^ active α5β1 integrin distribution on the EC surface. b.l., basolateral. (**c**) Quantitative analysis of apico-basal mean intensity ratio of SNAKA51^+^ active α5β1 integrin. SNAKA51^+^ active α5β1 integrin localizes on the basolateral surface of pLVX siCTL ECs, but not pLVX siPPFIA1 ECs in which it randomly redistributes all around the cell surface. Data are mean±s.e.m., *n*=20 cells per condition pooled from two independent experiments. Scale bar, 5 μm (**a**) ***P<0.001; Student's *t*-test.

Unlike non-polarized fibroblasts^42^, FN fibrils are deposited beneath the basolateral surface of polarized ECs^17^,^18^,^19^. Similarly to what has been previously reported for active αvβ3 integrin after shear stress^43^, quantitative analysis of apico-basal mean intensity ratio by confocal xz sectioning on both sparse (Supplementary Fig. 3) and confluent ECs (Fig. 3a–c) revealed that SNAKA51^+^/active α5β1 integrins localize along the basolateral side of pLVX siCTL ECs, while in pLVX siPPFIA1 ECs they were randomly redistributed around the cell surface (Fig. 3a–c). Also in this case, lentiviral transduction of PPFIA1r rescued the defective polarized membrane distribution of SNAKA51^+^/active α5β1 integrin caused by PPFIA1 silencing in ECs (Fig. 3a–c). Furthermore, confocal xz sectioning demonstrated that also PPFIA1-GFP resides along the basolateral side of confluent ECs (Supplementary Fig. 4).

Taken together, these data indicate that in ECs PPFIA1 selectively associates with and regulates the trafficking of SNAKA51^+^/active α5β1 integrins.

### PPFIA1 controls FN secretion and polymerization

**Figure 4:**
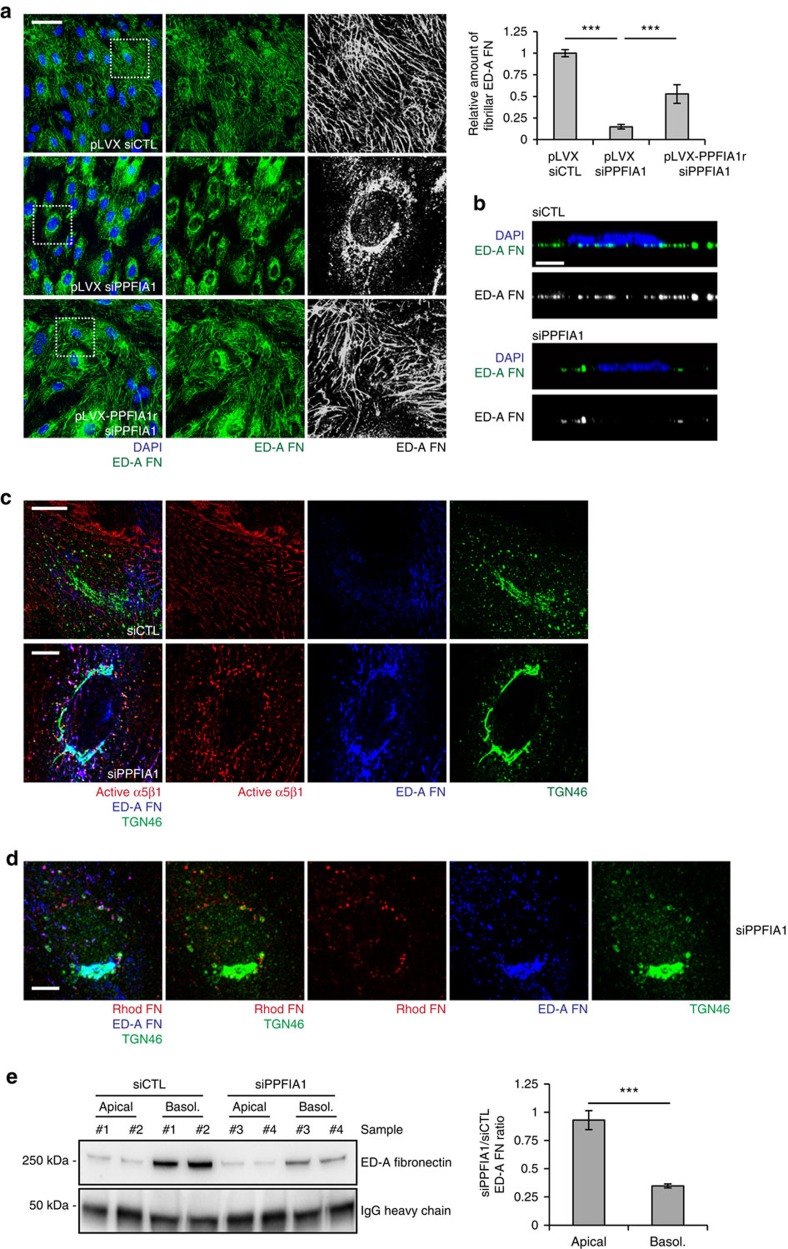
PPFIA1 controls endogenous ED-A FN exit from the TGN and its polymerization underneath confluent ECs. (**a**) Confocal microscopy analysis of IST9 mAb-labelled endogenous ED-A FN (green) in confluent ECs. ED-A FN polymerizes into a fibrillar network in pLVX siCTL, but not in pLVX siPPFIA1 ECs in which it accumulates in perinuclear punctae. PLVX-mediated PPFIA1r overexpression restores ED-A FN polymerization in siPPFIA1 ECs. The relative amount of fibrillar ED-A FN area was calculated in pLVX siCTL, pLVX siPPFIA1 and pLVX-PPFIA1r+siPPFIA1 ECs. Data are mean±s.e.m., *n*=20 cells per condition pooled from two independent experiments. (**b**) Confocal xz sections of ED-A FN (green) in siCTL or siPPFIA1 ECs. Compared with siCTL ECs, the basolateral accumulation of polymerized ED-A FN decreases dramatically in siPPFIA1 ECs. (**c**) Confocal microscopy characterization of the perinuclear compartment in which ED-A FN accumulates on PPFIA1 silencing in ECs. Before fixation, living ECs were incubated for 20 min with exogenous SNAKA51. Compared with siCTL ECs, ED-A FN (blue) heavily accumulates in the TGN46^+^ (green) TGN cisternae of siPPFIA1 ECs. Of note, in siPPFIA1 ECs exogenously added SNAKA51 mAb (red) binds active α5β1 integrins that, on endocytosis, reach TGN46^+^ post-Golgi carrier vesicles, but do not enter in TGN cisternae. (**d**) Fluorescent confocal microscopy shows that, similarly to SNAKA51, exogenously added rhodamine (Rhod, red) labelled serum FN reaches TGN46^+^ (green) post-Golgi carrier vesicles, but does not enter in TGN cisternae of siPPFIA1 ECs in which endogenous ED-A FN (blue) heavily accumulates instead. (**e**) Western blot analysis of soluble ED-A FN released by confluent ECs seeded on Transwell inserts. An equal percentage of apical and basolateral volumes of medium were collected after 72 h of culture from different wells of siCTL or siPPFIA1 ECs. Equal amounts of exogenous rabbit IgG were added to samples (spike normalization) for loading control purposes. Quantification of the ratio between apical or basolateral amount of ED-A FN released by siCTL over siPPFIA1 ECs. PPFIA1 silencing impairs basolateral, but not apical ED-A FN secretion. Data are mean±s.e.m., *n*=8 wells per condition pooled from 4 independent experiments. Scale bar, 50 μm (**a**), 10 μm (**c**,**d**), 5 μm (**b**). ***P<0.001; Student's *t*-test.

Since α5β1 integrin is the principal FN receptor in ECs and its localization in fibrillar adhesions is fundamental to funnel actomyosin tension necessary to unfold and incorporate secreted FN dimers into polymeric fibrils^8^, we asked whether the polymerization of extra domain-A (ED-A)-containing cellular FN (ED-A FN) was controlled by PPFIA1. Confluent pLVX siCTL, pLVX siPPFIA1 and pLVX-PPFIA1r siPPFIA1 ECs were cultured in medium containing plasma FN-depleted fetal bovine serum (FBS) and endogenous ED-A FN was visualized with the IST9 mAb and confocal fluorescence microscopy. While pLVX siCTL ECs polymerized ED-A FN into an extracellular fibrillar network (Fig. 4a, upper panels), which confocal xz sectioning localized to the basolateral membranes (Fig. 4b), pLVX siPPFIA1 ECs accumulated FN in a perinuclear compartment inside ECs (Fig. 4a, middle panels). Importantly, pLVX-mediated PPFIA1r overexpression strongly decreased ED-A FN intracellular accumulation and restored its polymerization (Fig. 4a, lower panels). Addition of exogenous FN to the medium did not rescue the defective fibrillogenesis of endogenous ED-A FN in PPFIA1-silenced ECs (Supplementary Fig. 5).

Next, we investigated whether the subcellular compartment in which ED-A FN accumulated on PPFIA1 silencing overlapped with the compartment(s) in which active α5β1 integrins accumulated in siPPFIA1 ECs. To this end, living cells were incubated with SNAKA51 mAb at 37 °C for 20 min, fixed, stained and analyzed by fluorescence confocal microscopy. Interestingly, in contrast to pLVX siCTL ECs, pLVX siPPFIA1 ECs displayed a large accumulation of ED-A FN in TGN46^+^ TGN cisternae. Furthermore, ED-A FN staining overlapped with internalized SNAKA51 mAb-bound, active α5β1 integrin only in TGN46^+^ vesicular PGCs, but not in TGN cisternae of pLVX siPPFIA1 ECs (Fig. 4c). Moreover, exogenously added rhodamine-labelled plasma FN reached the TGN46^+^ positive PGCs of siPPFIA1 ECs, but did not enter the TGN cisternae where instead endogenous ED-A FN accumulated (Fig. 4d). Next, we explored if PPFIA1 silencing affects not only the assembly of ED-A FN into a basolateral fibrillar meshwork, but also its polarized secretion. To this end, we set up a quantitative biochemical apico-basal secretion assay that, through the use of Transwell polycarbonate membrane inserts, allowed quantification of the amount of soluble ED-A FN that confluent ECs released in either the apical or basolateral culture medium. In keeping with previous findings^17^,^18^,^19^, we observed that the secretion of endogenous ED-A FN mainly occurred at the basolateral side of ECs (Fig. 4e). Notably, PPFIA1 silencing severely impaired basolateral, but not apical, ED-A FN secretion in the medium of confluent cultured ECs (Fig. 4e).

**Figure 5:**
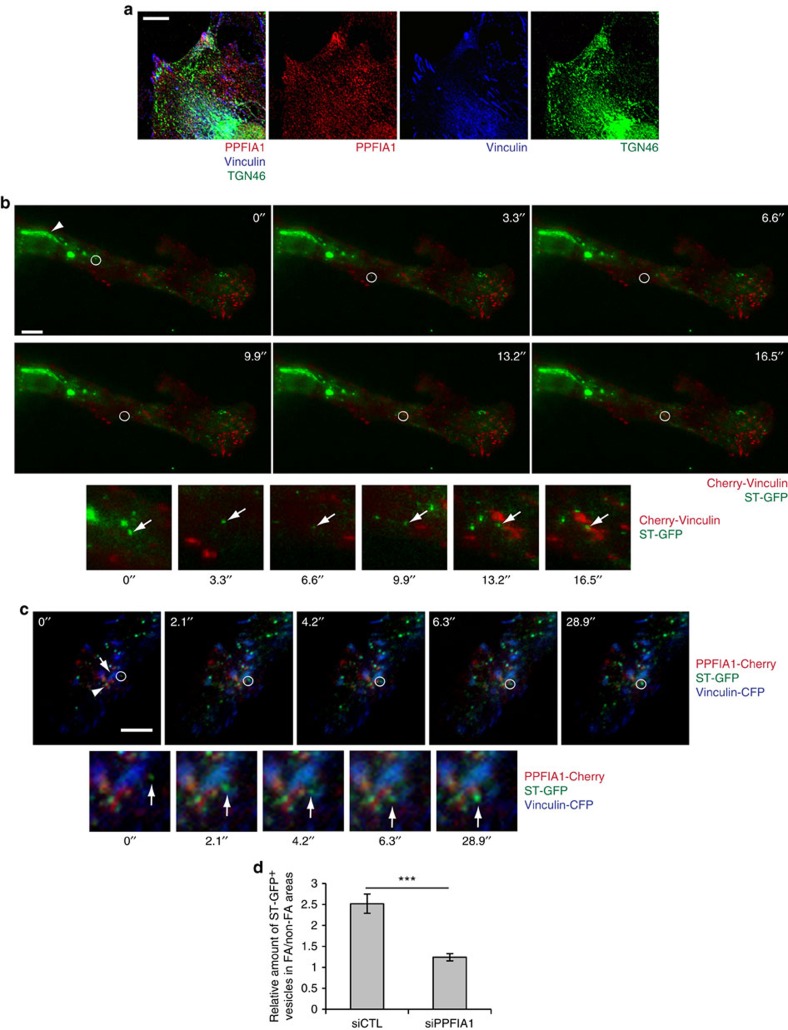
Post-Golgi carrier vesicles target EC adhesion sites. (**a**) Confocal microscopy analysis on fixed ECs shows that, in addition to labelling the perinuclear TGN cisternae, TGN46 (green) is present in post-Golgi carrier vesicles that preferentially accumulate, together with PPFIA1 (red), around vinculin-containing (blue) adhesion sites. (**b**) Snapshots from live TIRF microscopy of a region of an EC that was co-transfected with the TGN marker ST-GFP (green) and the adhesion site component Cherry-vinculin (red). ST-GFP is clearly localized at the TGN cisternae (white arrowhead) and in peripheral post-Golgi vesicles, the trajectory of one of which (white circle) is tracked from the perinuclear area to a peripheral Cherry-vinculin-labelled adhesion site. Single channel photograms are shown in Supplementary Fig. 3. (**c**) Snapshots from live TIRF microscopy of a region of an EC that was co-transfected with ST-GFP (green), PPFIA1-Cherry (red) and the vinculin-CFP (blue). The trajectory of one ST-GFP post-Golgi vesicles (white circle) is tracked in proximity of a CFP-vinculin labelled adhesion site (white arrow), where it reaches a PPFIA1-Cherry^+^ area (white arrowhead) and resides for at least 12 s. More in general, several ST-GFP-labelled vesicles closely associate to PPFIA1 surrounded vinculin-containing adhesion sites (Supplementary Movies 1 and 2). Single channel photograms are shown in Supplementary Fig. 3. (**b**,**c**) Lower panels are magnifications of the white circled areas shown in the above panels. White arrows indicate representative ST-GFP^+^ PGCs that are reaching PPFIA1-Cherry^+^ areas. (**d**) Quantification of the relative amount of ST-GFP vesicles residing in focal adhesion containing (FA) or non-containing (non-FA) areas in siCTL or siPPFIA1 ECs. Data are mean±s.e.m., *n*=10 cells per condition pooled from three independent experiments. Scale bar, 10 μm (**a**–**c**). ***P<0.001; Student's *t*-test.

Altogether, these data show how in ECs the lack of PPFIA1 simultaneously impairs the recycling of endocytosed active α5β1 integrin and the basolateral secretion of endogenous ED-A FN. Furthermore, PPFIA1 silencing results in an abnormal accumulation of both ED-A FN and endocytosed active α5β1 integrin in PGCs. With respect to the role of PPFIA1 in the docking of secretory vesicles to the plasma membrane of neuronal presynaptic terminals^31^,^32^, we asked whether TGN46^+^ PGCs might also reach the basolateral plasma membrane of ECs and localize in close proximity to adhesion sites where PPFIA1 concentrates (Fig. 1; ref. 33). Confocal microscopy analysis on fixed ECs revealed a sizeable accumulation of TGN46^+^ PGC vesicles around vinculin-containing adhesion sites where PPFIA1 also localizes (Fig. 5a). To evaluate further the possible targeting of PGCs in close proximity (100 nm) to adhesion sites, we performed time-lapse total internal reflection fluorescence (TIRF) microscopy on living ECs co-transfected with the GFP-tagged TGN marker sialyltransferase (ST-GFP, gift of Julia von Blume) and the Cherry-tagged adhesion site component vinculin (Cherry-vinculin, gift of Kenneth Yamada). ST-GFP localized in typical perinuclear TGN cisternae (Fig. 5b, white arrowhead), as well as in more peripheral vesicular PGCs, several of which were observed targeting close to Cherry-vinculin-containing adhesion sites (Supplementary Movie 1; Fig. 5b; Supplementary Fig. 6a). Similarly, TIRF microscopy revealed that ST-GFP^+^ PGCs are also delivered in close proximity to PPFIA1-Cherry-positive structures (Supplementary Movie 2; Fig. 5c; Supplementary Fig. 6b) that concentrate around vinculin-CFP-containing ECM adhesions (Fig. 1; ref. 33). Quantitative analyses of snapshots from live time-lapse TIRF microscopy unveiled in siCTL, but not in siPPFIA1 ECs, a statistically significant preferential targeting of ST-GFP^+^ PGCs in proximity of Cherry-vinculin-labelled ECM adhesions, as opposed to Cherry-vinculin-devoid non-adhesive plasma membrane regions (Fig. 5d).

To sum up, when PPFIA1 is absent, endocytosed active α5β1 integrins accumulate in PGCs and integrin recycling to the EC surface slows down. Furthermore, on PPFIA1 silencing, a significant fraction of endogenous ED-A FN arrests in the TGN and fails to polymerize. Thus, PPFIA1 controls the recycling of internalized active α5β1 integrin as well as the polarized basolateral secretion and polymerization of newly synthesized endogenous ED-A FN in ECs.

### Fibrillar adhesions and FN fibrils are dynamic structures

**Figure 6:**
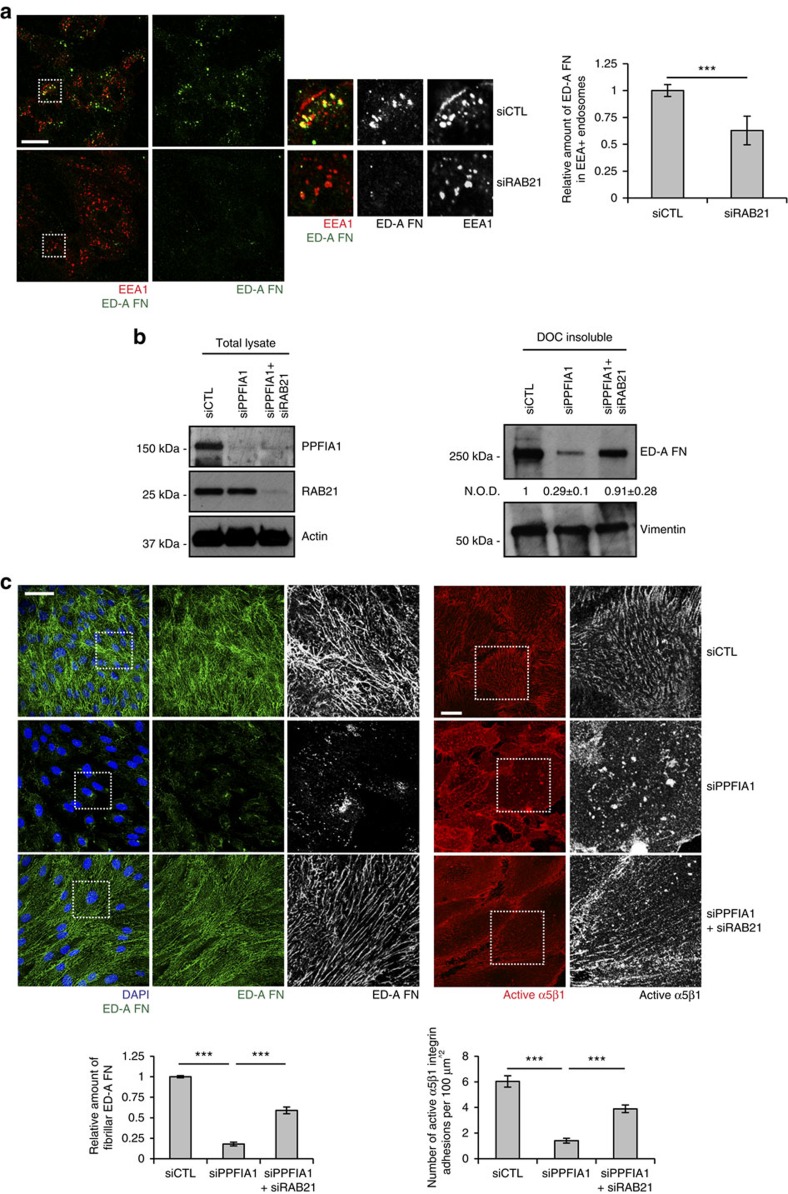
Fibrillar adhesions and FN fibrils are dynamic structures governed by Rab21 and PPFIA1. (**a**) Living siCTL and siRAB21 ECs were incubated with IST9 mAb for 30 min, fixed, acid washed and stained. RAB21 silencing impairs ED-A FN endocytosis as revealed by the decrease of IST9 punctae co-localizing with the endosome marker EEA1. Right panels are magnifications of the boxed areas in left panels. Data are mean value±s.e.m., *n*=70 cells per condition pooled from two independent experiments. ***P<0.001. (**b**) Left panel, western blot analysis of PPFIA1, RAB21 and actin on total lysates of siCTL, siPPFIA1 and siPPFIA1+siRAB21 ECs. Right panel, western blot analysis of the insoluble matrix fraction of ECs that were extracted with DOC buffer. PPFIA1 silencing dramatically reduces the amount of DOC-insoluble fraction of endogenous ED-A FN. Of note, simultaneous silencing of Rab21 GTPase (siPPFIA1+siRAB21), which drives integrin endocytosis, rescues the defective incorporation of endogenous ED-A FN in the DOC-insoluble fraction of siPPFIA1 ECs. (**c**) Confocal microscopy analysis of the patterning of endogenous cellular ED-A FN (green) in fixed confluent ECs. Before fixation, living ECs were incubated for 20 min with exogenous SNAKA51 (red). ED-A FN polymerizes into a fibrillar network in siCTL, but not in siPPFIA1 ECs. Simultaneous silencing of Rab21 GTPase (siPPFIA1+siRAB21) fully restores ED-A FN polymerization in siPPFIA1 ECs. SNAKA51^+^ active α5β1 integrin localizes in fibrillar adhesion in siCTL, but not in siPPFIA1 ECs. Notably, simultaneous Rab21 (siPPFIA1+siRAB21) silencing promotes the localization of SNAKA51 in fibrillar adhesion of siPPFIA1 ECs. Data are mean±s.e.m., *n*=20 cells per condition pooled from two independent experiments. Scale bar, 20 μm (**a**,**c**, right), 50μm (**c**, left). ***P<0.001; Student's *t*-test.

Since impairing the polarized basolateral secretion of newly synthesized ED-A FN diminishes FN fibrillogenesis (Fig. 4a), we hypothesized that the presence of the FN network underneath confluent ECs depends on its continuous turnover, which in turn hinges on a fine balance between endocytosis-mediated removal of old and cleaved FN fragments and exocytosis-mediated deposition of new FN dimers. To explore this hypothesis directly, we sought to impair the endocytosis of active α5β1 integrins from fibrillar adhesions and determine how this affects FN fibril formation in PPFIA1-silenced ECs. To interfere with active α5β1 integrin internalization, we focused on the small GTPase Rab21, which has been reported to control active β1 integrin endocytosis^44^,^45^ and localizes both on early endosomes and at the TGN^46^. We found Rab21 to control the endocytosis of endogenous ED-A FN in ECs (Fig. 6a).

Western blots of deoxycholate (DOC) buffer-extracted insoluble ED-A FN from confluent siCTL, siPPFIA1 and siPPFIA1+siRAB21 ECs revealed that PPFIA1 silencing dramatically reduced the amount of DOC-insoluble ED-A FN in ECs, while simultaneous silencing of PPFIA1 and Rab21 (siPPFIA1+siRAB21) rescued the defective incorporation of endogenous ED-A FN in the DOC-insoluble fraction of siPPFIA1 ECs (Fig. 6b). Confocal microscopy showed that, compared with siCTL ECs (Fig. 6c, top left panels), ED-A FN failed to polymerize into a fibrillar network in siPPFIA1 ECs (Fig. 6c, middle left panels), while simultaneous silencing of Rab21 GTPase (siPPFIA1+siRAB21) fully restored ED-A FN polymerization in siPPFIA1 ECs (Fig. 6c, bottom left panels). Moreover, fluorescence confocal microscopy analysis revealed that, after incubation on confluent living ECs for 20 min at 37 °C, SNAKA51^+^/active α5β1 integrin localized to fibrillar adhesion in siCTL (Fig. 6c, top right panels) , but not in siPPFIA1 ECs (Fig. 6c, middle right panels). Importantly, simultaneous PPFIA1 and Rab21 (siPPFIA1+siRAB21) silencing reestablished the localization of SNAKA51 in fibrillar adhesions of siPPFIA1 ECs (Fig. 6c, bottom right panels).

Thus, the plasticity of fibrillar adhesions and the deposition of FN fibrils depend on the functional connection of Rab21 and PPFIA1 that respectively signal to control the endocytosis and recycling/exocytosis of active α5β1 integrin and cellular ED-A FN.

### PI4KB/AP-1A/PTPRF-driven FN release and active α5β1 traffic

**Figure 7:**
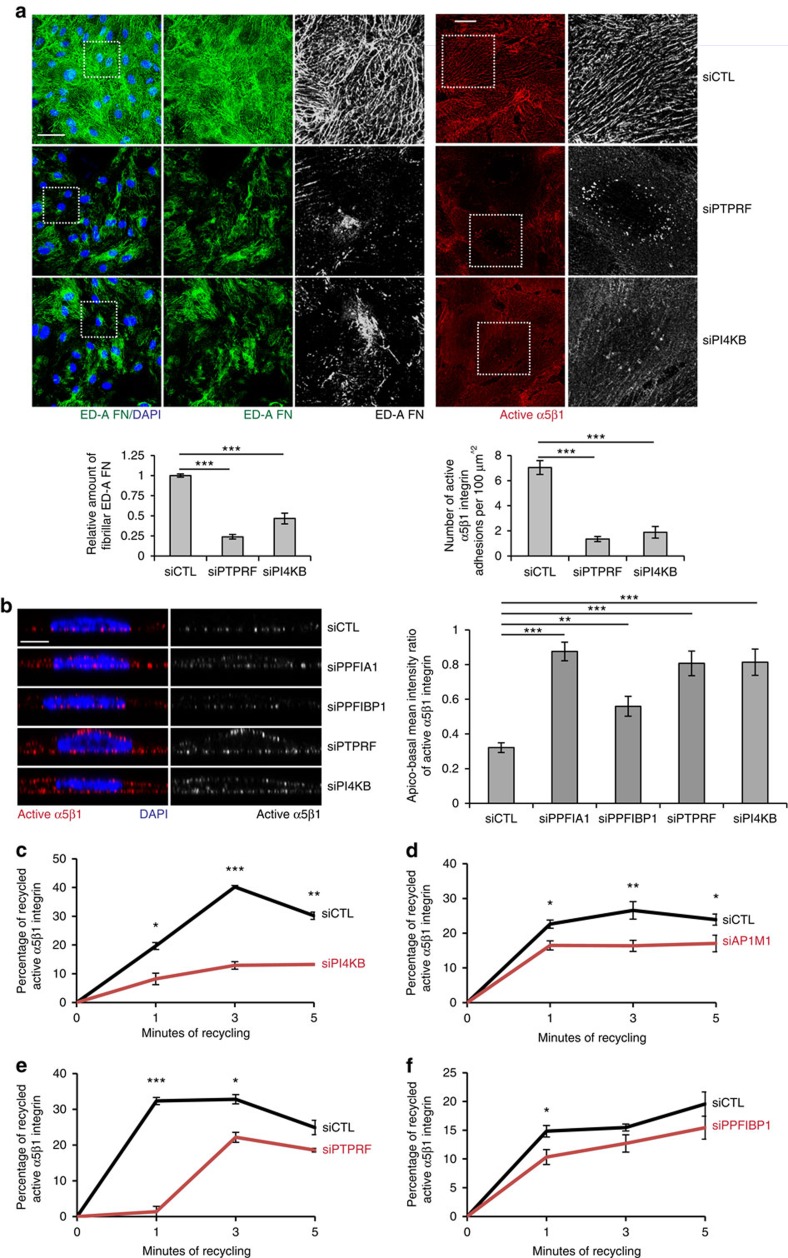
PTPRF cooperates with PI4KB and AP-1A to control ED-A FN exit from the TGN and polymerization as well as active α5β1 integrin recycling. (**a**) Confocal microscopy analysis of the patterning of endogenous cellular ED-A FN (green) in fixed confluent ECs and anti-active α5β1 integrin mAb SNAKA51 (red) incubated on living confluent ECs for 20 min before fixation. ED-A FN polymerizes into a fibrillar network in siCTL, but neither in siPI4KB nor in siPTPRF ECs. SNAKA51^+^ active α5β1 integrin localizes in fibrillary adhesion in siCTL, but neither in siPI4KB nor in siPTPRF ECs. Relative amount of fibrillary ED-A FN area was measured in siCTL, siPTPRF and siPI4KB ECs. Data are mean±s.e.m., *n*=20 cells per condition pooled from two independent experiments. The number of active α5β1 integrin-containing adhesions per 100 μm^2^ of cell area was quantified in siCTL, siPTPRF and siPI4KB ECs. Data are mean±s.e.m., *n*=20 cells per condition pooled from two independent experiments. (**b**) Quantitative analysis of apico-basal mean intensity ratio of SNAKA51^+^ active α5β1 integrin by confocal xz sectioning on confluent ECs. SNAKA51^+^ active α5β1 integrin localizes on the basolateral surface of siCTL, but neither siPPFIA1, nor siPTPRF, nor siPI4KB ECs in which it randomly redistributes all around the cell surface. Silencing of PPFIBP1 affects the apico-basal distribution of SNAKA51^+^ active α5β1 integrin much less efficiently. (**c**–**f**) Time-course analysis of the relative amounts of recycled active α5β1 integrin in siCTL ECs versus siPPFIBP1 or siPTPRF or siAP1M1 or siPI4KB ECs, as evaluated by integrin recycling assay and capture ELISA assay. Silencing of either PTPRF or PI4KB or AP1M1 significantly impairs the recycling of SNAKA51^+^ active α5β1 integrin. Data are mean±s.e.m., *n*=3 independent experiments (two technical replicates for each experiment). Scale bar, 50 μm (**a**, left), 20 μm (**a**, right), 5 μm (**b**). *P<0.05; **P<0.01; ***P<0.001; Student's *t*-test.

To identify proteins involved in the recycling of endocytosed active α5β1 integrin in ECs, we investigated the role of crucial regulators of PGC biogenesis^21^, such as phosphatidylinositol 4-kinase, catalytic, beta (PI4KB) and the EC-expressed clathrin adaptor protein complex-1A (AP-1A). In addition, we characterized the function of PTPRF that directly binds^28^ and cooperates with PPFIA1 in the docking of TGN-derived neurotransmitter vesicles to the active zone of neuronal presynaptic nerve terminals^31^,^32^. To this end, we examined by confocal microscopy the deposits of endogenous cellular ED-A FN in confluent ECs silenced with siCTL or a pool of four siRNA directed against either PTPRF (siPTPRF) or PI4KB (siPI4KB) (Supplementary Fig. 7). Endogenous ED-A FN polymerized into a fibrillar network in siCTL (Fig. 7a, top left panels), but neither in siPTPRF (Fig. 7a, middle left panels) nor in siPI4KB (Fig. 7a, lower left panels) ECs. Similarly, SNAKA51^+^/active α5β1 integrin localized in fibrillar adhesion of siCTL, but not in siPTPRF or siPI4KB ECs (Fig. 7a, right panels). Quantitative analysis of apico-basal mean intensity ratio of SNAKA51^+^/active α5β1 integrin by confocal xz sectioning on confluent ECs revealed that SNAKA51^+^/active α5β1 integrins localized on the basolateral surface of siCTL. However, in siPPFIA1, siPTPRF, or siPI4KB ECs SNAKA51^+^/active α5β1 integrins redistributed randomly all around the cell surface (Fig. 7b). For control purposes, we silenced the liprin β protein PPFIBP1, that similarly to PPFIA1 binds PTPRF and control synaptic size^30^, but whose function in organizing the traffic of presynaptic nerve terminals is poorly understood^29^. We found that PPFIBP1 silencing only modestly affected the apico-basal distribution of SNAKA51^+^/active α5β1 integrin in ECs (Fig. 7b). Furthermore, silencing of either PI4KB (Fig. 7c) or the key AP-1A subunit AP1M1 (ref. 47) (siAP1M1) (Fig. 7d) or PTPRF (Fig. 7e) significantly impaired the recycling of SNAKA51^+^/active α5β1 integrin. As in the case of active α5β1 integrin apico-basal distribution, knocking down PPFIBP1 affected the recycling of SNAKA51^+^/active α5β1 integrin much less efficiently (Fig. 7f).

Altogether, these data indicate that, similarly to PPFIA1 (Fig. 4), PI4KB, AP-1A and PTPRF control the recycling of active α5β1 integrin as well as the polymerization of a FN fibrillar network underneath cultured ECs. Furthermore, the data show that the internalized active α5β1 integrins exploit the same PI4KB/AP-1A-dependent trafficking pathway for recycling.

### Rab11B drives FN secretion and active α5β1 recycling

**Figure 8:**
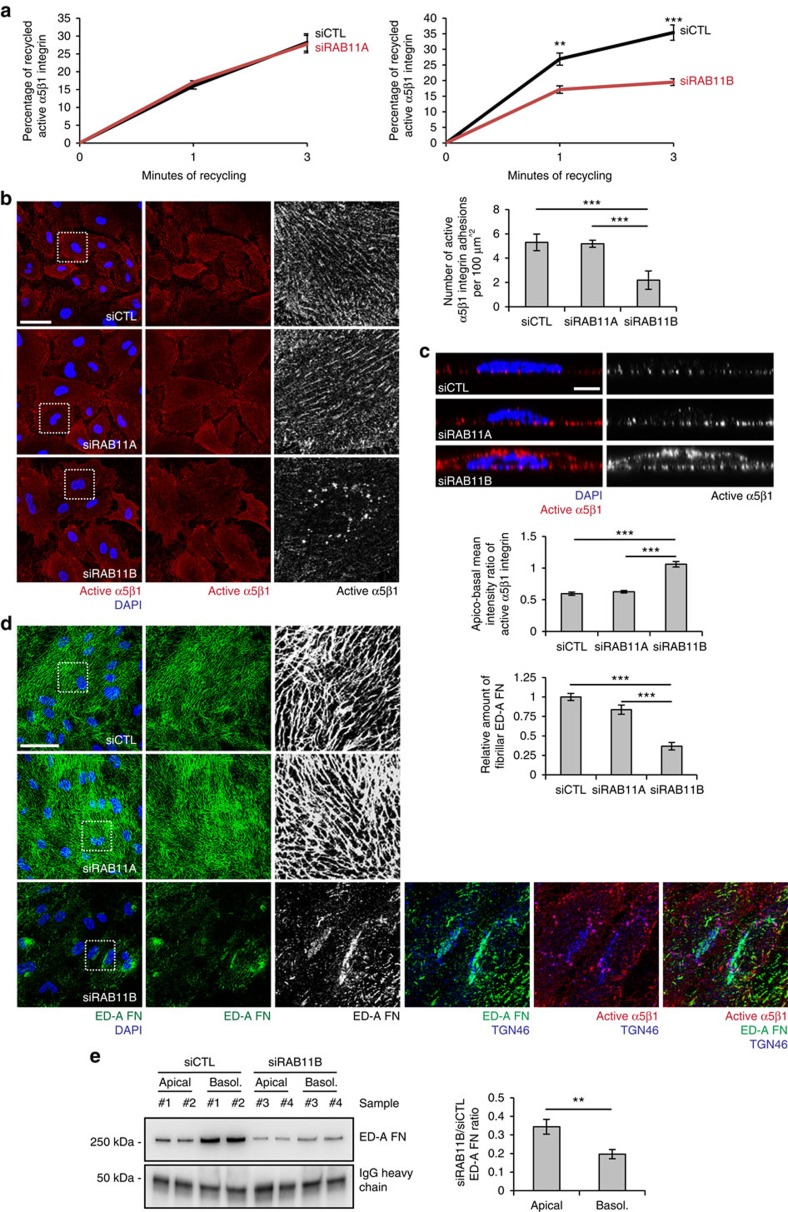
RAB11B controls the recycling of endocytosed active α5β1 integrins and polarized ED-A FN secretion in ECs. (**a**) Time-course analysis of recycled active α5β1 integrin in siCTL ECs versus siRAB11A ECs or siRAB11B ECs. RAB11B, but not RAB11A, silencing significantly impairs active α5β1 integrin recycling. Data are mean±s.e.m., *n*=3 independent experiments (two technical replicates for each experiment). (**b**) Confocal microscopy analysis of anti-active α5β1 integrin mAb SNAKA51 localization (red) in living confluent ECs (20 min of incubation). SNAKA51^+^ active α5β1 integrin localizes in fibrillar adhesion in siCTL and in siRAB11A, but not in siRAB11B ECs in which it accumulates in perinuclear punctae. The number of active α5β1 integrin-containing adhesions per 100 μm^2^ of cell area was quantified in siCTL, siRAB11A and siRAB11B ECs. Data are mean values±s.e.m., *n*=20 cells per condition pooled from two independent experiments. (**c**) Confocal xz sectioning microscopy analysis of anti-active α5β1 integrin mAb SNAKA51 localization (red) in living confluent ECs (20 min of incubation). Quantitative analysis of apico-basal mean intensity ratio reveals that SNAKA51^+^ active α5β1 integrin localizes on the basolateral surface of siCTL and siRAB11A, but not of siRAB11B ECs, where it redistributes all around the cell surface. Data are mean values±s.e.m., *n*=20 cells per condition pooled from two independent experiments. (**d**) Confocal microscopy analysis of IST9 mAb^+^ endogenous cellular ED-A FN (green) in confluent ECs. ED-A FN polymerizes into a fibrillar network in siCTL and siRAB11A, but not in siRAB11B ECs, where it accumulates in a perinuclear compartment. Relative amount of fibrillary ED-A FN area was calculated in siCTL, siRAB11A and siRAB11B ECs. Data are mean values±s.e.m., *n*=20 cells per condition pooled from 2 independent experiments. (**e**) Western blot analysis of soluble ED-A FN released by confluent ECs seeded on Transwell inserts. An equal percentage of apical and basolateral volumes of medium were collected after 72 h of culture from different wells of siCTL or siRAB11B ECs. Equal amounts of rabbit IgG were exogenously added to samples (spike normalization) for loading control purposes. Quantification of the ratio between apical or basolateral amount of ED-A FN released by siCTL over siRAB11B ECs. RAB11B silencing much more severely impairs basolateral than apical ED-A FN secretion. Data are mean±s.e.m., *n*=6 wells per condition pooled from three independent experiments. Scale bar, 50 μm (**d**), 20 μm (**b**), 5μm (**c**). **P<0.01; ***P<0.001; Student's *t*-test.

The results above indicated how in ECs, the same molecular and cellular mechanisms control active α5β1 integrin recycling as well as the basolateral secretion and polymerization of ED-A FN. Rab11 small GTPases, which localize at recycling endosomes, TGN and PGCs^48^, regulate the slow recycling of active β1 integrins^13^,^39^. Next, we investigated if in ECs Rab11 proteins may simultaneously orchestrate active α5β1 integrin recycling together with ED-A FN secretion and fibrillogenesis. We found that ECs express both Rab11A and Rab11B proteins (Supplementary Fig. 7). In polarized epithelial cells, Rab11A resides in a vesicular compartment distinct from that of Rab11B^49^ and, Rab11A, but not Rab11B, controls the apical delivery of post-Golgi cargoes^50^. When compared with siCTL ECs, Rab11B (siRab11B), but not Rab11A (siRab11A) knockdown halved the amount of recycled active α5β1 integrin in ECs (Fig. 8a). Similarly, on incubation on living confluent ECs, SNAKA51 mAb largely localized in α5β1-containing fibrillar adhesions of siCTL and siRab11A, but not siRab11B ECs (Fig. 8b). In addition, quantitative confocal xz sectioning analysis on confluent ECs revealed that although SNAKA51^+^/active α5β1 integrins localize along the basolateral side of siCTL and siRab11A ECs, they redistributed randomly around the surface of siRab11B ECs (Fig. 8c). In addition, while siCTL and siRab11A ECs polymerized ED-A FN into an extracellular fibrillar network, siRab11B ECs accumulated FN in a perinuclear compartment, consisting of TGN46^+^ TGN cisternae (Fig. 8d). Moreover, in siRab11B ECs SNAKA51^+^/active α5β1 integrins accumulated in perinuclear TGN46^+^ PGCs (Fig. 8d). Finally, quantitative biochemical apico-basal secretion assays revealed that Rab11B silencing preferentially impaired basolateral ED-A FN secretion in the medium of confluent cultured ECs (Fig. 8e). We hence infer that in ECs the Rab11B small GTPase orchestrates the recycling of endocytosed active α5β1 integrins together with the basolaterally-polarized secretion and polymerization of endogenous ED-A FN.

### α5β1 controls FN secretion and polymerization

**Figure 9:**
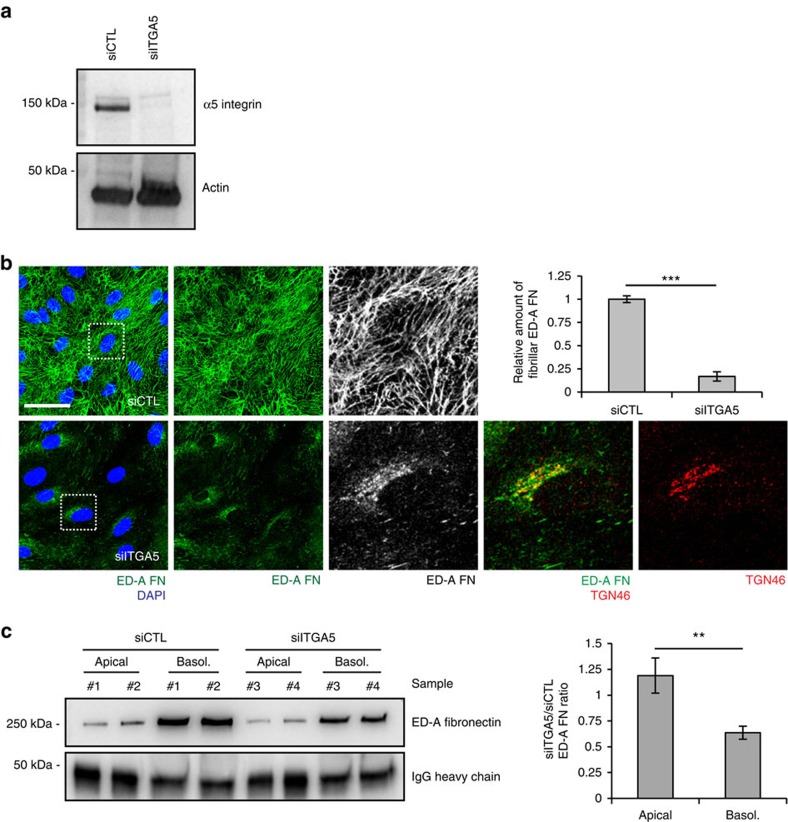
α5β1 regulates ED-A FN secretion and polymerization. (**a**) Western blot analysis of lysates of ECs control (siCTL) and α5 integrin subunit (siITGA5) silenced ECs. Cells were lysed 24 hours after the second siRNA oligofection and proteins were separated by SDS–PAGE and probed for α5 integrin subunit or actin (for control purposes). (**b**) Confocal microscopy analysis of IST9 mAb-labelled endogenous ED-A FN (green) in confluent ECs. ED-A FN polymerizes into a fibrillar network in siCTL, but not in siITGA5 ECs in which it accumulates in the TGN46+ (red) TGN cisternae. The relative amount of fibrillar ED-A FN area was calculated in siCTL and siITGA5 ECs. Data are mean±s.e.m., *n*=20 cells per condition pooled from two independent experiments. ***P<0.001; Student's *t*-test. (**c**) Western blot analysis of soluble ED-A FN released by confluent ECs seeded on Transwell inserts. An equal percentage of apical and basolateral volumes of medium were collected after 72 h of culture, from different wells of siCTL or siITGA5 ECs. Equal amounts of exogenous rabbit IgG were added to samples (spike normalization) for loading control purposes. Quantification of the ratio between apical or basolateral amount of ED-A FN released by siCTL over siITGA5 ECs. α5 integrin subunit silencing impairs basolateral, but not apical ED-A FN secretion. Data are mean±s.e.m., *n*=8 wells per condition pooled from four independent experiments. **P<0.01; Student's *t*-test. Scale bar, 50 μm (**b**).

Altogether, the above results led us to postulate that α5β1 integrin may play a key direct role in the control of ED-A FN secretion. To verify this hypothesis, we investigated whether interfering with α5β1 integrin function in ECs may indeed hamper the secretion of ED-A FN from the TGN and its assembly into a basolateral fibrillar network. Therefore, to hinder directly α5β1 integrin function, we silenced the α5 integrin subunit (siITGA5) in ECs (Fig. 9a). Of note, in addition to the expected severe impairment of FN fibrillogenesis, the silencing of α5 integrin subunit also promoted a clear accumulation of endogenous ED-A FN in the TGN of ECs (Fig. 9b). Furthermore, quantitative biochemical apico-basal secretion assays unveiled that α5 integrin subunit silencing significantly decreases the basolateral, but not apical secretion of ED-A FN in the medium of confluent cultured ECs (Fig. 9c). Thus, blocking α5β1 integrin function in ECs inhibits the basolateral secretion of endogenous ED-A FN from the TGN.

### PPFIA1 drives vascular morphogenesis *in vitro* and *in vivo*

Polarized FN secretion and matrix polymerization are required for vascular morphogenesis *in vitro*^7^ and for vessels formation *in vivo* in the developing mouse embryo^1^. First, we directly tested the influence of PPFIA1 on vascular morphogenesis *in vitro* by means of EC capillary formation assays in growth factor-reduced Matrigel. After 8 h incubation at 37 °C, pLVX siCTL ECs formed capillary networks surrounded by a dense meshwork of polymerized cellular ED-A FN. By contrast, pLVX siPPFIA1 ECs failed to form a fibrillar FN network and remained immobile on the Matrigel surface without forming cell-to-cell contacts and a capillary network. Importantly, pLVX-mediated PPFIA1r re-expression rescued FN network formation, EC mobility and the formation of capillary networks (Supplementary Fig. 8).

**Figure 10:**
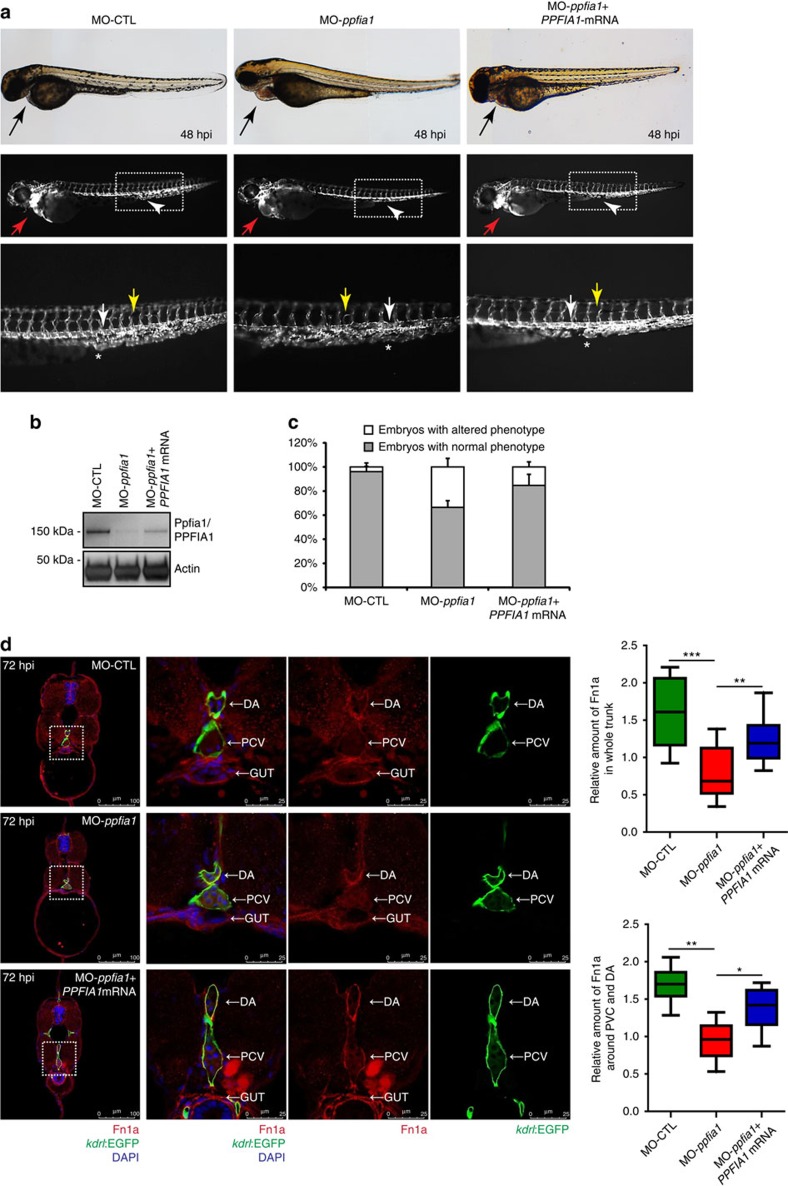
PPFIA1 silencing affects vascular morphogenesis in developing zebrafish embryo. (**a**) Lateral view at conventional light and confocal fluorescence microscopy of *Tg(kdrl:EGFP)* zebrafish embryos carrying EC-specific EGFP expression and derived from fertilized zebrafish eggs that were injected with 83 μM of a *ppfia1* translation blocking morpholino (MO-*ppfia1*), 5-base mismatch (MO-CTL), or *ppfia1* translation blocking morpholino and 40 pg *PPFIA1* mRNA at the single-cell stage. Starting from 48 hpi, ∼35% of the ppfia1 morphants displayed: (i) an enlarged heart chamber phenotype (red arrows) due to atrium dilation associated with pericardial oedema, blood stasis and reduced blood flow despite the presence of cardiac activity; (ii) malformations in the intersegmental vessels (Se, yellow arrow); (iii) an irregular shape (margin) in the DA and the PCV (white arrows). Human *PPFIA1* mRNA co-injection successfully rescued the cardiovascular defects in the vast majority (∼84%) of *ppfia1* morphants that appeared morphologically normal (**b**). Western blot analysis of MO-*ppfia1*, MO-CTL and MO-*ppfia1+PPFIA1* mRNA morphants, at 48 hpi, revealed that the band corresponding to zebrafish ppfia1 present in MO-CTL, completely disappear in MO-ppfia1 and it is partially restored in MO-*ppfia1+PPFIA1* mRNA morphants. Actin was used as loading control. (**c**) Relative percentage of the embryos having normal or altered phenotype in two independent experiments is reported in the graph and both absolute numbers with relative percentage of the embryos for each experimental group are reported in Supplementary Tables 1 and 2. (**d**) Confocal fluorescence microscopy analysis of MO-CTL, MO-*ppfia1* and MO-*ppfia1+ PPFIA1* mRNA *Tg(Kdrl:EGFP)* zebrafish embryos trunk cross-sections at 72 hpi stained with DAPI and Fn1a. Right panels are magnifications of boxed areas of the left panels. While MO-CTL morphants show normal Fn1a expression around the PCV, DA, gut and in the whole trunk cross-section (upper panels); MO-*ppfia1* morphants display a decreased Fn1a expression in the overall trunk region (middle panels). Co-injection of human *PPFIA1* mRNA in MO-*ppfia1* morphants rescued Fn1a deposition defects albeit not completely in the overall trunk cross-section. Relative amount of Fn1a expression in MO-CTL, MO-*ppfia1* and MO-*ppfia1+ PPFIA1* mRNA *Tg(Kdrl:EGFP)* zebrafish whole trunk cross-sections or around PVC and DA, at 72 hpi. Data are mean values±s.d. from 4 (MO-CTL) or 9 (MO-*ppfia1* and MO-*ppfia1+ PPFIA1* mRNA) embryos from two independent experiments, and 5th/95th percentile.*P<0.05; **P<0.01; ***P<0.001; ANOVA followed by the Tukey range test. Scale bar, 100 μm (**d**, left panels), 25 μm (**d**, right panels).

Next, we assessed the effects of PPFIA1 inactivation *in vivo*. Antisense morpholino oligonucleotides (MOs) were used to knockdown Ppfia1 protein expression in the transgenic zebrafish line *Tg*(*kdrl:EGFP)* expressing EGFP in ECs. To this end, a translation blocking MOs (experimental group; MO-*ppfia1*) and the corresponding five-base mismatch MOs (control group; MO-CTL) were independently microinjected into fertilized zebrafish eggs at the single-cell stage. At 48 h post-injection (hpi), the gross appearance of *ppfia1* morphant embryos was normal. However, approximately 35% of them exhibited cardiovascular defects, such as an enlarged heart chamber phenotype due to atrium dilation associated with pericardial oedema, blood stasis (Fig. 10a), and reduced blood flow despite the presence of cardiac activity (Supplementary Movies 3 and 4). In contrast, control-injected embryos appeared phenotypically normal. Furthermore, sporadic haemorrhages in the subintestinal vascular plexus, malformations in the intersegmental vessels (Se) and irregular shapes (margin) of the dorsal aorta (DA) and the posterior cardinal vein (PCV) were also present in defective *ppfia1* morphants (Fig. 10a). At 72 hpi, all these phenotypic cardiovascular alterations persisted, but did not further exacerbate.

To exclude the possibility that cardiovascular aberrations of *ppfia1* morphants might be due to MO off-target effects, we assessed whether co-injecting human *PPFIA1* mRNA, which was not targeted by either *ppfia1* MOs, could rescue the observed phenotypes^51^. We analyzed wild type embryos, *ppfia1* morphants and *ppfia1* morphants co-injected with human *PPFIA1* mRNA, confirming its effective translation into the corresponding PPFIA1 protein by western blot, although at lower levels than that of endogenous Ppfia1 in wild type embryos (Fig. 10b). As previously observed, about 35% of 48 hpi *ppfia1* morphants exhibited the described cardiovascular defects, while control-injected embryos were unaffected. Human *PPFIA1* mRNA co-injection successfully rescued the cardiovascular defects in the large majority (∼84%) of *ppfia1* morphants that appeared morphologically normal (Fig. 10a,c), while a minor fraction of them (∼16%) still exhibited cardiovascular abnormalities.

Next, we directly evaluated whether the lack of Ppfia1 may affect the deposition of FN in the living zebrafish. To this end, we performed quantitative fluorescence confocal microscopy analyses of FN-immunostained trunk sections of 72 hpi wild type embryos, *ppfia1* morphants and *ppfia1* morphants co-injected with human *PPFIA1* mRNA. As shown in Fig. 10d, *ppfia1* morphants exhibited a sizeable reduction in FN deposition both around DA and PCV and in overall trunk sections (Fig. 10d). Co-injection of human *PPFIA1* mRNA in *ppfia1* morphants rescued FN deposition defects albeit not completely (Fig. 10d), suggesting a dose-dependent threshold for the different phenotypic alterations observed in *ppfia1* morphants (Fig. 10b).

In summary, PPFIA1 depletion inhibits endothelial FN secretion and polymerization, and impairs vascular morphogenesis in cultured ECs and in the developing zebrafish embryo.

## Discussion

The onset of cell polarity is a key event for organ development^52^ in multicellular organisms^53^. Polarized cells of epithelial tissues, nervous and vascular systems and invading cancers, are characterized by spatially and functionally defined membrane areas that originate from, and are maintained by, complexes of molecules dedicated either to the compartmentalization or to the directional targeting of their own structural components^22^. Once established, cells must coordinate the maintenance of polarity with the continuous turnover of transmembrane proteins and ECM at specific subcellular sites. In contrast to fibroblasts^42^, FN deposition is for example restricted to the basolateral surface of polarized ECs^17^.

In the case of epithelial cells^22^ and ECs^5^, polarization is defined by an apical and a basolateral plasma membrane area. It has been proposed that β1 integrins act as sensors that define and orient the apico-basal cell axis^4^,^5^,^6^. During embryonic cardiovascular development, basolateral FN secretion and assembly into a bioactive fibrillar network by EC-expressed β1 integrins are crucial for the establishment of endothelial apico-basal polarity and vascular morphogenesis^3^. Hence, β1 integrins may define the orientation of the apico-basal axis and at the same time convey positional information, allowing a continuous basally polarized secretion of newly synthesized FN and giving rise to a self-sustaining positive feedback loop. Here, we have investigated this possibility. We found that in ECs the cytoplasmic adaptor PPFIA1: (i) localizes close to α5β1-containing fibrillar adhesions; (ii) physically interacts with the cytodomain of active α5β1 integrin; (iii) controls the recycling to the cell surface of endocytosed active α5β1 integrin; (iv) drives the polarized distribution of active α5β1 integrin on the basolateral cell surface; (v) promotes the localization of PGCs near ECM adhesions. These observations, together with the fact that, on PPFIA1 silencing, active α5β1 integrin accumulates in PGCs, support a working model in which PPFIA1 allows the polarized recycling of active α5β1 integrin to fibrillar adhesions of the basolateral side of ECs by exploiting TGN-mediated secretory pathways and interacting with β1 integrin cytotails. On internalization into early endosomes, active α5β1 integrins, likely bound to protease-cleaved fragments of polymeric extracellular FN^9^,^10^, reach PGCs from where they may recycle to the EC basolateral surface along with newly synthesized FN dimers derived from the TGN cisternae (Supplementary Fig. 9). This cellular pathway represents an ideal strategy to orchestrate the removal of endocytosed and old FN with the deposition of newly synthesized FN allowing a continuous, but dynamic renewal of polarized FN fibrils. It is still unclear how and where the old, endocytosed FN fragments separate from active α5β1 integrins, and if the subpopulation of new FN dimers bind active α5β1 integrins in PGCs or if the recycling of endocytosed active α5β1 integrin is just providing a positional signal for the polarized FN secretion. Since integrins are bidirectional allosteric adhesion receptors^54^, in the absence of sizeable mechanical tension, freshly synthesized FN binding may be sufficient to maintain α5β1 integrin in active conformation within PGCs, around which we did not detect talin or kindlins. In any event, our observation that the silencing of α5 integrin subunit in ECs impairs the basolateral secretion of endogenous ED-A FN from the TGN supports the assumption that α5β1 integrin controls polarized FN exocytosis.

Central players in the turnover of active α5β1 integrin at EC fibrillar adhesion sites are PPFIA1 and PTPRF, which may provide the information to direct α5β1 integrin-containing carriers for basolateral integrin recycling, while remaining at the basal surface, where they probably define specific membrane regions. Interestingly, PTPRF and PPFIA1 exert a similar function in neuronal presynaptic areas where they compartmentalize structural components of the so-called active zone and mediate neurotransmitter vesicle docking^31^,^32^. PPFIA1 is also part of a multi-protein complex that localizes close to integrin adhesion sites to control cortical microtubule dynamics and targeting^33^. Since microtubules are essential for polarized protein and lipid traffic^55^, the microtubule-related function of PPFIA1 may represent an additional and complementary strategy by which it may favour basolateral targeting of PGCs containing recycling active α5β1 integrins and newly synthesized FN in ECs. Further work will determine if PPFIA1 triggers additional biochemical signal(s) promoting the basolateral secretion of freshly synthesized FN.

Accumulating evidence supports the notion that integrins and their ligands exhibit a high traffic-dependent turnover within adhesion sites^11^,^12^,^13^. Small GTPases belonging to the Rab5 subfamily, namely Rab5 and Rab21, control β1 integrin internalization^44^,^45^,^56^. Analogous to observations in fibroblasts^56^, we also found that endothelial fibrillar adhesions, which are considered the most mature α5β1 integrin-containing adhesive contacts^57^, are highly dynamic in nature. However, the turnover of fibrillar adhesions in fibroblasts does not appear to be significantly influenced by the master regulator of early endocytosis Rab5 (ref. 56). Instead, we propose a model where active α5β1 integrin-containing fibrillar adhesions of ECs and associated FN fibrils rely on the coupling of Rab21-dependent internalization and PPFIA1-driven recycling or exocytosis from the TGN of their adhesive receptor or ligand components respectively (Supplementary Fig. 9).

The role that regulators of PGC biogenesis, such as PI4KB and AP-1 (refs 20, 21, 22), play in active α5β1 integrin recycling suggests that, through the TGN, the biosynthetic routes connect to the endosomal paths that traffic these ECM receptors back and forth from adhesion sites. In this regard, it will be important to determine in the future whether receptors other than integrins may exploit trafficking to the TGN to convey signals modulating the biosynthetic routes according to the presence, amount and location of extracellular cues in polarized cells (Supplementary Fig. 9). In this respect, along the biosynthetic routes of epithelial cells, transmembrane proteins can be either directly routed from the TGN to the plasma membrane or trafficked through recycling endosomal compartments before reaching the apical or basolateral surface^22^. However, it is unknown if recycling endosomal compartment may be also involved in the polarized secretion of soluble proteins, such as FN. Here, we reveal how in ECs the regulator of slow endosomal recycling Rab11B coordinates with inducers of PGC formation, that is AP1M1 and PI4KB, and the plasma membrane-associated vesicle tethering complex PTPRF/PPFIA1 to control the basolateral secretion of endogenous ED-A FN in conjunction with the recycling of endocytosed active α5β1 integrins (Supplementary Fig. 9).

FN is required for developmental blood vessel^1^,^3^ and heart morphogenesis both in mice^3^ and in zebrafish^58^. Indeed, FN and its major receptor α5β1 integrin regulate blood vessel lumen formation^4^,^7^ and the acquisition of a polarized epithelial organization by migrating myocardial progenitors^58^,^59^. The depletion of *ppfia1 in vivo* in the developing zebrafish embryo caused severe heart morphogenesis defects resembling those observed in *nat*/*fn* mutants^58^, impairs perivascular FN deposition, and the patterning of trunk large vessels. This data, together with the defects in EC migration, vessel formation and FN polymerization *in vitro* emphasizes the relevance of the molecular network capable of coordinating polarized active α5β1 integrin recycling and FN exocytosis in cardiovascular cells.

## Methods

### Antibodies and reagents

Rabbit polyclonal anti-PPFIA1 was from Proteintech Group, Chicago, IL, USA (characterized on siCTL and siPPFIA1 ECs in Supplementary Fig. 10). Goat polyclonal anti-Nrp1 (C-19), mouse monoclonal anti-ED-A FN (clone IST9) and goat polyclonal anti-EEA1 (N-19) were from Santa Cruz Biotechnology, Dallas, TX, USA. Rabbit polyclonal anti-α5-integrin (AB1949), mouse monoclonal anti-kindlin-2 (Mab2617) and mouse monoclonal anti-Myc (05-419) were from Merk Millipore, Billerica, MA, USA. Mouse monoclonal anti-vinculin (V9131), anti-Flag-M2-HRP (A8592) and anti-α tubulin (T5168) were from Sigma Aldrich, St Louis, MO, USA. Mouse monoclonal anti-actin (NB100-74340) was from Novus Biologicals, Littleton, CO, USA. Mouse monoclonal anti-LAMP1 (555798) was from BD Biosciences. Rabbit polyclonal anti-TGN46 (AHP1586) and sheep polyclonal anti-TGN46 (AHP500) were from AbD Serotec, Oxford, UK. Rabbit polyclonal anti-RAB11A (TA324158) and RAB11B (TA346586) were from Origene, Rockville, MD, USA. Mouse monoclonal anti-active-α5-integrin, SNAKA51, was previously described^34^. Rabbit polyclonal anti-Rab21 was kindly gifted from Johanna Ivaska, Turku Centre for Biotechnology, University of Turku, Finland. Antigen, provider and dilution used in different experimental settings for all the commercial and non-commercial primary antibodies employed in this study are summarized in Supplementary Table 3.

Goat anti-rabbit (sc-2054) and donkey anti-goat (sc-2056) HRP secondary antibodies were from Santa Cruz Biotechnology, while goat anti-mouse (115-035-003) HRP secondary antibody was from Jackson ImmunoResearch Laboratories, West Grove, PA, USA. Alexa Fluor 555 goat anti-mouse IgG2a (A21137), Alexa Fluor 488 goat anti-mouse IgG1 (A21121), Alexa Fluor 647 (A31571), 488 (A21202) and 555 (A31570) donkey anti-mouse IgG, Alexa Fluor 555 donkey anti-rabbit (A31572), Alexa Fluor 647 donkey anti-goat (A21447), Alexa Fluor 488 donkey anti-sheep (A11015) secondary antibodies, DAPI (D3571) and TO-PRO3 (T3605) were from Life technologies, Carlsbad, CA, USA.

### Recombinant proteins

Human plasma FN (1918-FN-02M) was from R&D Systems, Minneapolis, MN, USA. Rhodamine-labelled bovine FN (FNR01-A) was from Cytoskeleton, Denver, CO, USA. Cellular ED-A FN isolated from human foreskin fibroblasts, (F2518) was from Sigma Aldrich.

### Cell culture

Primary arterial ECs were isolated from the umbilical cords as previously described^14^. Briefly, umbilical artery was cannulated with a blunt 17-gauge needle and the needle was secured by clamping the cord over the needle. The artery was perfused with 50 ml of PBS to wash out the blood. A total of 10 ml of 0.2% collagenase A (Cat. No. 11088793001, Roche Diagnostics, Risch-Rotkreuz, Switzerland) in cell culture medium was then infused into the umbilical artery and incubated 30 min at room temperature. The collagenase solution containing the ECs was flushed from the cord by perfusion with 40 ml of PBS, collected in a sterile 50 ml centrifuge tube and centrifuged 5 min at 800 *g*. Cells were resuspended in Endothelial Cell Growth Basal Medium (EBM-2) supplemented with EGM-2 BulletKit (Lonza Basel, Switzerland) (EGM-2) and plated in cell culture dishes adsorbed with 1% gelatin from porcine skin (G9136, Sigma Aldrich). Cells were tested for mycoplasma contamination by means of Venor GeM Mycoplasma Detection Kit (MP0025-1KT, Sigma Aldrich) and grown in EGM-2. The isolation of primary arterial ECs human umbilical cords was approved by the Office of the General Director and Ethics Committee of the Azienda Sanitaria Ospedaliera Ordine Mauriziano di Torino hospital (protocol approval no. 586, Oct 22 2012 and no. 26884, Aug 28 2014) and informed consent was obtained from each patient.

### DNA constructs

Human PPFIA1 cDNA (transcript variant 1, NM_003626) from Origene Technologies Rockville, MD, USA was used as a template. PPFIA1r mutant construct was obtained by PCR, according to the Taq polymerase manufacturer's instructions, using Phusion Site-Directed Mutagenesis Kit (Life technologies). Forward primers, containing two silent point mutation on each sequence targeted by siRNA, are:

Fw1_AAGAAAGGTTGCGACAAATGGAAGCAC;

Fw2_GAACGACACTGGCGTATGGGGACATG;

Fw3_GATCGAGTGATACGGTGGATCCTGTC;

Fw4_AGGTGAGAGAACGATTGCGAGTAGCAC.

Afterwards, mutants were subcloned in pLVX-IRES-Puro lentiviral vector (Clontech, Mountain View, CA, USA), using standard PCR protocols, using Phusion High-Fidelity DNA Polymerase (Life technologies). Lentiviral particles were produced as described in the manufacturer's guidelines. pmCherry Vinculin was a gift from Kenneth Yamada, NIH, Bethesda, MD (Addgene plasmid #50527). ST-GFP was generated by Dr Ian Trowbridge^60^ and kindly provided by Julia Von Blume, Max Planck Institute for Biochemistry, Martinsried, Germany.

### Gene silencing

The day before oligofection, ECs were seeded in six-well dishes at a concentration of 10 × 10^4^ cells per well. Oligofection of siRNA duplexes was performed according to the manufacturer's protocols. Briefly, human ECs were transfected twice (at 0 and 24 h) with 200 pmol of siCONTROL nontargeting siRNA (as control), siGENOME SMART pools siRNA oligonucleotides (GE, Dharmacon, Lafayette, CO, USA), using Oligofectamine Transfection Reagent (Life technologies). 24 h after the second oligofection, ECs were lysed or tested in functional assays.

### Immunoprecipitation and western blotting

ECs were lysed in a buffer containing 25 mM Tris–HCl, pH 7.6, 100 mM NaCl, 0.15% Tween-20, 5% glycerol, 0.5 mM ethylene glycol tetraacetic acid (EGTA) and Protease Inhibitor Cocktail (Sigma Aldrich), 2 mM phenylmethanesulfonylfluoride (PMSF), 2 mM MgCl_2_. Cell lysates were incubated for 20 min on wet ice, and then centrifuged at 15,000 *g*, 20 min, at 4 °C. The total protein amount was determined using the bicinchoninic acid (BCA) protein assay reagent (Life technologies). Equivalent amounts (800 μg) of protein were immunoprecipitated for 1 h with the antibody of interest, and immune complexes were recovered on Protein G-Sepharose (GE Healthcare). Immunoprecipitates were washed twice with lysis buffer, twice with the same buffer without Tween-20 and then separated by SDS–PAGE. Proteins were then transferred to a nitrocellulose membrane (Biorad, Hercules, CA, USA), probed with antibodies of interest and detected by an enhanced chemiluminescence technique (PerkinElmer, Waltham, MA, USA).

Uncropped scans of all blots are provided as Supplementary Figs 11–14.

### Pull-down assays

For bacterial expression, the intracellular domain of β1 integrin (β1-cyto; residues from 752 to 799 of the full length mouse protein) or α5 integrin (α5-cyto; residues from 1,026 to 1,054 of the full length mouse protein) were cloned into the pET15b vector (Clontech) in frame with the dimerization domains of Fos (residues from 161 to 200 of full length mouse protein) and Jun (residues from 277 to 318 of full length mouse protein) respectively. The 6 × His tag sequence in pET15b vector (Clontech) was replaced with 3 × Flag tag and Myc tag to generate Flag-Jun-α5 cyto, Flag-Fos-β1 cyto and Myc-Jun-α5 cyto constructs.

Constructs were transformed into BL21 (DE3) Arctic Express Escherichia coli (Agilent Technologies, Santa Clara, CA, USA) and protein expression was induced with 0.8 mM IPTG for 3 h at 30 °C. Afterwards, bacteria were pelleted by centrifugation 1485 *g*, 4 °C, resuspended in TBS buffer (50 mM Tris pH 7.4, 150 mM NaCl) containing Lysozyme 100 μg ml^−1^ and DNase 50 μg ml^−1^ and rotated for 2 h at 4 °C. After the addition of Empigen (30% solution) (1 ml per 10 ml of lysate) the bacterial lysates were rotated for 1 h at 4 °C and centrifuged 1,485 *g* for 1 h at 4 °C. Flag-Jun-α5, Flag-Fos-β1 and a mixture of Flag-Jun-α5 and Myc-Fos-β1 protein supernatants were incubated with ANTI-FLAG M2 Affinity Gel (Sigma, A2220) 2 h at 4 °C followed by extensive washing of the beads three times with TBS buffer. In parallel, mouse fibroblast cell lysate were lysed by for 10 min on ice with lysis buffer (50 mM Tris-HCl (pH 7.5), 150 mM NaCl, 1% Triton X-100 and protease inhibitors (Roche, Basel, Switzerland), phosphatase inhibitor cocktail 2 and 3 (Sigma) and cleared by centrifugation. Overall, 1 mg of cell lysate was added to the beads and incubated for 2 h at 4 °C with gentle agitation. After several washes with lysis buffer, proteins were subjected to SDS–PAGE and western blotting with indicated antibodies.

### Integrin recycling assay

Integrin recycling assays were performed as previously described by Roberts *et al*.^38^ with minor modifications. Cells were transferred to ice, washed twice in cold phosphate-buffered saline (PBS) and surface-labelled at 4 °C with 0.2 mg ml^−1^ sulfo-NHS-SS-biotin (Life technologies) in PBS for 30 min. Labelled cells were washed in cold PBS and transferred to prewarmed EGM-2 at 37 °C. After 5, 10 or 15 min, the medium was aspirated, and dishes were rapidly transferred to ice and washed twice with ice-cold PBS. Biotin was removed from proteins remaining at the cell surface by incubation with a solution containing 20 mM sodium 2-mercaptoethanesulfonate (MesNa) in 50 mM Tris-HCl (pH 8.6), 100 mM NaCl for 1 h at 4 °C. MesNa was quenched by the addition of 20 mM iodoacetamide (IAA) for 10 min, and after ECs were reicubated at 37 °C for the indicated times. Afterwards, the medium was aspirated, and dishes were rapidly transferred to ice and washed twice with ice-cold PBS, biotin was removed from proteins recycled at the cell surface with MesNa and MesNa was quenched by IAA. ECs were lysed in 25 mM Tris–HCl, pH 7.4, 100 mM NaCl, 2 mM MgCl_2_, 1 mM Na_3_VO_4_, 0.5 mM EGTA, 1% Triton X-100, 5% glycerol, Protease Inhibitor Cocktail (Sigma Aldrich) and 1 mM PMSF. Lysates were cleared by centrifugation at 12,000 *g* for 20 min. Supernatants were corrected to equivalent protein concentrations and levels of biotinylated integrin were determined by capture ELISA assay.

### Capture ELISA assay

Corning 96 Well Clear Polystyrene High Bind Stripwell Microplate (product #2592, Corning, Corning, NY, USA) were coated overnight with 5 μg ml^−1^ appropriate anti-integrin antibodies in 0.05 M Na_2_CO_3_ (pH 9.6) at 4 °C and were blocked in PBS containing 0.05% Tween-20 (PBS-T) with 5% BSA for 1 h at RT. Integrins were captured by overnight incubation of 50 μl cell lysate at 4 °C. Unbound material was removed by extensive washing with PBS-T, and wells were incubated with streptavidin-conjugated horseradish peroxidase (GE Healthcare) in PBS-T containing 1% BSA for 1 h at 4 °C. Following further washing, biotinylated integrins were detected by a chromogenic reaction with ortho-phenylenediamine.

### Conventional and g-STED confocal scanning microscopy

Cells were plated on 0.17 mm glass coverslips (no. 1.5) coated with 1% gelatin (in PBS) or 3 μg ml^−1^ FN and allowed to adhere overnight. Cells were washed in PBS, fixed in 4% paraformaldehyde (PFA), permeabilized in 0.1% Triton X-100 for 2 min on ice, incubated with different primary antibodies for 1 h at RT, and revealed by appropriate Alexa-Fluor-tagged secondary antibody (Life Technologies). Cells were analyzed by using a Leica TCS SP2 AOBS confocal laser-scanning microscope (Leica Microsystems, Wetzlar, Germany). A Leica TCS SP8 gated stimulated emission depletion (g-STED) 3 × laser-scanning microscope was used to acquire super-resolved images (Leica Microsystems). A Leica STED HC PL APO 100 × /1.40 objective was used. Fluorochromes and fluorescent proteins were excited at the optimal wavelength by means of 80 MHz pulsed white light laser (470–670 nm), allowing time gating of fluorescence life times. For STED, the appropriate, 592 or 660 nm, depletion laser was used. Fluorescence channels were scanned sequentially and emission was revealed by means of hybrid spectral detectors (HyD SP Leica Microsystems).

To quantify the localization of active α5β1 integrin in fibrillar adhesions, we counted the number of SNAKA mAb positive objects using Image J shape descriptor AR (aspect ratio), selecting only the ones displaying a major_axis/minor_axis. ratio ≥2 per 100 μm^2^ of cell area.

To quantify the apico-basal distribution of active α5β1 integrin, we measured mean fluorescence intensity for membrane length unit of SNAKA51 antibody for apical and basolateral membrane measuring total fluorescence intensity on the two membranes using Image J quantification tool and dividing it for relative membrane length. We reported in the histogram the ratio between apical and basolateral mean fluorescence intensity. Image acquisition was performed by adopting a laser power, gain and offset settings that allowed maintaining pixel intensities (grey scale) within the 0–255 range and hence avoided saturation.

### Internalization assays

Cells were plated on 0.17 mm glass coverslips (no. 1.5) coated with 1% gelatin (in PBS) or 3 μg ml^−1^ FN and allowed to adhere overnight. Cells were incubated either with anti-active α5 integrin subunit SNAKA51 mAb or anti-ED-A FN IST9 mAb for 20 min at 37 °C, then washed with PBS and incubated 0.5 M NaCl, 0.5% acetic acid, 2.6 pH for 6 min at 4 °C to remove the antibodies bound on the cell surface. Cells were then washed thrice with PBS, fixed with 4% PFA and processed for immunofluorescence. The amount of internalized active α5β1 integrin or ED-A FN was quantified as mean fluorescence intensity of SNAKA51 or IST9 antibodies in EEA positive or TGN46 positive structures by means of Image J software.

### FN fibrillogenesis

Small interfering RNA silencing was performed, and, after the second oligofection, cells were seeded onto gelatin coated 0.17 mm glass coverslips (no. 1.5) in 24-well dishes at a concentration of 15 × 10^4^ cells per well or in 6-well dishes at a concentration of 4 × 10^5^ cells per well and left to adhere for 16 h in EGM-2 medium containing FN-depleted serum. Cells on coverslips were incubated with SNAKA51 mAb for 20 min at 37 °C, then washed with PBS, fixed with 4% PFA and processed for immunofluorescence. The amount of ED-A FN fibrils was quantified as the fraction of cell area stained by IST9 mAb by means of Image J software.

Cells plated in 6-well dishes were either processed for sodium deoxycholate (DOC) solubility assay or lysed in hot SDS lysis buffer to analyze protein expression by western blot. DOC solubility assay was performed as previously described^61^ with minor modifications. Briefly, ECs were put on ice and lysed with cold DOC lysis buffer (2% DOC; 20 mM Tris–HCl, pH 8.8; 2 mM PMSF; 2 mM EDTA; 2 mM iodoacetic acid; 2 mM N-ethylmaleimide). EC lysates were collected with a 26G needled syringe and immediately centrifuged at 18,800 *g*, for 15 min at 4 °C. The supernatant was removed; the pellet was washed once with DOC buffer and solubilized in 25 μl of SDS buffer (1% SDS; 20 mM Tris–HCl, pH 8.8; 2 mM PMSF; 2 mM EDTA; 2 mM iodoacetic acid; 2 mM N-ethylmaleimide). Protein concentration in DOC-soluble fractions was estimated using BCA protein assay, and used to normalize protein concentration in the corresponding DOC-insoluble fraction. DOC-insoluble samples were resolved by SDS–PAGE using 7.5% polyacrylamide gel, transferred to nitrocellulose and immunoblotted with IST9 mAb.

### Apico-basal FN secretion assay

ECs were seeded onto gelatin coated polycarbonate membrane Transwell inserts, pore 0.4 μm (3413, Corning, Lowell, MA, USA), at a 15 × 10^4^ cells per well density and left to adhere for 24 h in EGM-2 medium. Next, transendothelial Resistance (TER) was measured with Millicell ERS-2 Volt-Ohm Meter (Millipore) to verify endothelial monolayer integrity and the medium was replaced with EGM-2 containing FN-depleted serum (200 μl in the upper chamber and 600 μl in the lower chamber). After 72 h, the medium was collected and cleared by centrifugation at 15,000 *g*, 20 min, at 4 °C. Spike normalization was performed adding 5 μg of rabbit anti-mouse IgG in the samples collected from upper and lower chambers and visualized in western blot for loading control purpose. Equal percentage of apical and basolateral volume of medium were resolved by SDS–PAGE using 7.5% polyacrylamide gel, transferred to nitrocellulose, and immunoblotted with IST9 mAb or anti-rabbit IgG-HRP.

### Time-lapse TIRF microscopy

TIRF microscopy on living ECs was performed using a Leica AM TIRF MC system mounted on a Leica AF 6000LX workstation. Cells were plated onto glass-bottom dishes (WillCo-dish; WillCo Wells B.V., Amsterdam, The Netherlands) and placed onto a sample stage within an incubator chamber set to 37 °C, in an atmosphere of 5% CO_2_, 20% humidity. A Leica HC PL APO 63 × /1.47 NA oil-immersion objective was used, and laser penetration depth was set at 90 nm. Excitation and analysis of fluorescent proteins were performed with a 488 nm (for GFP) and 532 nm (for Cherry) laser. Imaging was recorded on a Hamamatsu EM-CCD camera (C9100-02, Hamamatsu, SZK, Japan).

### *In vitro* angiogenesis assay

Overall, 17,000 ECs were resuspended in 350 μl of 10% FBS EBM-2, plated in each well of a 48-well plate previously coated with 150 μl per well of 8 mg per ml growth factor-reduced Matrigel (BD Biosciences, Franklin Lakes, NJ, USA) and incubated at 37 °C, 5% CO_2_ in a humidified atmosphere for 8 h to allow vascular network formation. Samples were observed by a Leica DMI 3000 B inverted microscope (Leica Microsystems). Images were acquired with a CoolSNAP HQ camera (Photometrics, Tucson, AZ, USA) controlled by Image Pro Plus software (Media Cybernetics, Rockville, MD, USA).

### Generation and analyses of zebrafish morphants

The adults and embryo fishes were raised and maintained under standard laboratory conditions. Experimental procedures related to fish manipulation followed previously reported recommendations^62^ and conformed to the Italian regulations for protecting animals used in research, including DL 116/92. The Ethics committee of the University of Torino approved this study. Larvae were anesthetized and, then, sacrifice by ice chilling.

The transgenic zebrafish line (*Tg(Kdrl:GFP)*^*s843*^) carrying endothelial-specific expression of the EGFP, was used in the described experiments. Morpholinos were injected in the zebrafish eggs at single-cell stage using a CO_2_-based Picospritzer-III device and the phenotype assessed after 48 hpi. Knockdown of PPFIA1 was achieved through the microinjection of the following morpholino: 5′-TGGTGGGCATCACCTCGCACATCAT-3′. The following morpholino was used as PPFIA1 5- base mismatch control: 5′-TCGTGGCCATCAACTCGAACATAAT-3′. Morpholinos were synthesized by Genetools and resuspended in nuclease-free water. The mixture of injection was prepared diluting each morpholino in fish water and phenol red 5% to a final concentration of 83 μM. The volume of injection was set to 4 nl. The coding DNA sequence of PPFIA1 was PCR amplified and cloned into pCS2^+^ plasmid between ClaI and XhoI sites. The corresponding mRNA was prepared using an SP6 MessageMachine kit (Ambion) according to the manufacturer's indications and its size verified by gel electrophoresis. The *in vitro* synthesized capped mRNA was resuspended in nuclease-free water to a final concentration of 100 ng μl^−1^. For the rescue experiments, the mixture of injection was prepared diluting the PPFIA1 morpholino and PPFIA1 mRNA in fish water and phenol red 5% respectively to a final concentration of 83 μM and 10 ng μl^−1^. The volume of injection was set to 4 nl.

For the Western blot analysis 50 embryos for each experimental group were placed in a pre-chilled 3.5 cm petri dish containing ice-cold PBS with the addition of proteases and phosphatases inhibitors (Roche). The yolk sac was removed passing each embryo several times through a 200 μl tip. Deyolked embryos were subsequently transferred into new Eppendorf tubes and lysed in 2% SDS lysis buffer at 90 °C for 10 min. DNA was removed by sonication of the lysates with 3 pulses of 30 sec each. Lysates were then centrifuged for 15 min at 14,000g and protein concentration was determined with a detergent-compatible Lowry assay (DC Protein Assay, BioRad). Equal amounts of protein were resolved by SDS–PAGE using 7.5% polyacrylamide gel, transferred to nitrocellulose, and immunoblotted with the indicated antibodies.

For the immunofluorescence detection of Fn1a, Tg(kdrl:EGFP) zebrafish embryos were collected at 72 hpi, fixed overnight in 4% PFA at 4 °C, washed twice in PBS-Tween 0.1%, embedded in 4% low-melting agarose and therefore sectioned with a Leica vibratome to produce transversal cross-sections of 250 μm. After permeabilization with PBS-Tween 0.1%, the sections were washed three times and incubated overnight at 4 °C with a rabbit primary antibody (Sigma Aldrich F3648) directed against Fn1a (1:200 dilution in PBS-Tween 0.1%). The sections were washed three times in PBS-Tween 0.1% and therefore incubated O/N at 4 °C with a secondary anti-rabbit antibody (Life Technologies) conjugated with Alexa Fluor-568 (1:1,000 dilution in PBS-Tween 0.1%) and with 0.1% DAPI to counterstain the nuclei. After extensive washing in PBS-Tween 0.1%, the sections were washed once in dH_2_O and mounted on glass slides with Vectashield (Vector Labs).

Zebrafish sections were imaged with a Leica TCS SP5 X confocal microscope equipped with a tandem scanning system with an oil-immersion objective HCX PL FLUOTAR 40 × (NA 1.25) and Leica LAS AF software. Images were acquired after sequential excitation at 405 nm for DAPI, 488 nm for GFP and 543 nm for Alexa Fluor-568 followed by emission detection at the appropriate wavelength using a frame resolution of 1,024 × 1,024 pixels, a pinhole opening of 1 Airy and a scanning frequency of 200 Hz. For each section, z-stacks were acquired with 2 μm distance. The z-stacks were processed and analyzed by ImageJ. Background subtraction was performed using the rolling ball procedure set to 50 pixels and the pictures converted to 32-bit format. The integrated intensity for the three separated channels was calculated in both the total cross section and in the perivascular region surrounding the PCV and the DA vessels. The normalized FN values were calculated dividing the integrated intensity of the red channel (FN-1) for the integrated intensity of the blue channel (DAPI). The normalized ratio was plotted in Graphpad as box-wiskers plot.

### Statistical analysis

For statistical evaluation, results were analyzed by a two-tailed heteroscedastic Student's *t*-test.

For the zebrafish embryo, experimental data were visualized by box-whisker plot showing the lower quartile (bottom box), the upper quartile (top box), median (band near the middle of the box), the fifth percentile (lower end of whisker) and the ninety-fifth percentile (upper end of whisker). For multiple comparisons, the data were analyzed with a one-way analysis of variance (ANOVA) followed by the Tukey range test. Stars indicate the following *P*-values: *P<0.05; **P<0.01; ***P<0.001. Neither randomization nor blinding was applied for zebrafish embryo analyses.

### Data availability

The authors declare that all data supporting the findings of this study are available within the article and its Supplementary Information files or are available from the corresponding author upon request.

## Additional information

**How to cite this article:** Mana, G. *et al*. PPFIA1 drives active α5β1 integrin recycling and controls fibronectin fibrillogenesis and vascular morphogenesis. *Nat. Commun.* **7**, 13546 doi: 10.1038/ncomms13546 (2016).

**Publisher's note**: Springer Nature remains neutral with regard to jurisdictional claims in published maps and institutional affiliations.

## Supplementary Material

Supplementary Information Supplementary Figures 1 - 14 and Supplementary Tables 1 - 3

Supplementary Movie 1 Live time-lapse total internal reflection fluorescence (TIRF) microscopy of an EC that was co-transfected with the TGN marker ST-GFP (green) and adhesion site protein Cherry-vinculin (red). ST-GFP is clearly localized at the TGN cisternae and in peripheral post-Golgi vesicles that very frequently target cherry vinculin enriched adhesion sites.

Supplementary Movie 2 Live time-lapse total internal reflection fluorescence (TIRF) microscopy of an EC that was co-transfected with the TGN marker ST-GFP (green), PPFIA1-Cherry (red) and adhesion site protein vinculin-CFP (blue). ST-GFP+ PGCs clearly target PPFIA1 enriched areas around vinculin containing adhesion sites.

Supplementary Movie 3 Live time-lapse confocal fluorescence microscopy of MO-*ppfia1* injected *Tg(kdrl:EGFP)* zebrafish embryo at 48 hpf. Time-lapse confocal fluorescence microscopy reveals normal heart beating in a representative 48 hpf MO-*ppfia1* injected *Tg(kdrl:EGFP)* zebrafish embryo.

Supplementary Movie 4 Live time-lapse phase contrast microscopy of MO-*ppfia1* injected *Tg(kdrl:EGFP)* zebrafish embryo at 48 hpf. Time-lapse phase contrast microscopy confirms normal heart beating in the same 48 hpf MO-*ppfia1* injected *Tg(kdrl:EGFP)* zebrafish embryo displayed in movie S3.

## References

[b1] Astrof S. & Hynes R. O. Fibronectins in vascular morphogenesis. Angiogenesis 12, 165–175 (2009). 19219555 10.1007/s10456-009-9136-6 PMC2716138

[b2] Weber G. F., Bjerke M. A. & DeSimone D. W. Integrins and cadherins join forces to form adhesive networks. J. Cell Sci. 124, 1183–1193 (2011). 21444749 10.1242/jcs.064618 PMC3115772

[b3] George E. L., Baldwin H. S. & Hynes R. O. Fibronectins are essential for heart and blood vessel morphogenesis but are dispensable for initial specification of precursor cells. Blood 90, 3073–3081 (1997). 9376588

[b4] Zovein A. C. . Beta1 integrin establishes endothelial cell polarity and arteriolar lumen formation via a Par3-dependent mechanism. Dev. Cell 18, 39–51 (2010). 20152176 10.1016/j.devcel.2009.12.006 PMC3178410

[b5] Iruela-Arispe M. L. & Davis G. E. Cellular and molecular mechanisms of vascular lumen formation. Dev. Cell 16, 222–231 (2009). 19217424 10.1016/j.devcel.2009.01.013 PMC8606173

[b6] Bryant D. M. & Mostov K. E. From cells to organs: building polarized tissue. Nat. Rev. Mol. Cell Biol. 9, 887–901 (2008). 18946477 10.1038/nrm2523 PMC2921794

[b7] Zhou X. . Fibronectin fibrillogenesis regulates three-dimensional neovessel formation. Genes Dev. 22, 1231–1243 (2008). 18451110 10.1101/gad.1643308 PMC2335318

[b8] Schwarzbauer J. E. & DeSimone D. W. Fibronectins, their fibrillogenesis, and *in vivo* functions. Cold Spring Harb. Perspect. Biol. 3, a005041 (2011). 21576254 10.1101/cshperspect.a005041 PMC3119908

[b9] Shi F. & Sottile J. MT1-MMP regulates the turnover and endocytosis of extracellular matrix fibronectin. J. Cell Sci. 124, 4039–4050 (2011). 22159414 10.1242/jcs.087858 PMC3244985

[b10] Shi F. & Sottile J. Caveolin-1-dependent {beta}1 integrin endocytosis is a critical regulator of fibronectin turnover. J. Cell Sci. 121, 2360–2371 (2008). 18577581 10.1242/jcs.014977 PMC2587120

[b11] Valdembri D. & Serini G. Regulation of adhesion site dynamics by integrin traffic. Curr. Opin. Cell Biol. 24, 582–591 (2012). 22981739 10.1016/j.ceb.2012.08.004

[b12] Jacquemet G., Humphries M. J. & Caswell P. T. Role of adhesion receptor trafficking in 3D cell migration. Curr. Opin. Cell Biol. 25, 627–632 (2013). 23797030 10.1016/j.ceb.2013.05.008 PMC3759831

[b13] De Franceschi N., Hamidi H., Alanko J., Sahgal P. & Ivaska J. Integrin traffic—the update. J. Cell Sci. 128, 839–852 (2015). 25663697 10.1242/jcs.161653 PMC4342575

[b14] Valdembri D. . Neuropilin-1/GIPC1 Signaling regulates α5β1 integrin traffic and function in endothelial cells. PLoS Biol. 7, e1000025 (2009). 10.1371/journal.pbio.1000025 PMC2631072 19175293

[b15] Rainero E. . Ligand-occupied integrin internalization links nutrient signaling to invasive migration. Cell Rep. 10, 398–413 (2015). 10.1016/j.celrep.2014.12.037 25600874

[b16] Dozynkiewicz M. A. . Rab25 and CLIC3 collaborate to promote integrin recycling from late endosomes/lysosomes and drive cancer progression. Dev. Cell 22, 131–145 (2012). 22197222 10.1016/j.devcel.2011.11.008 PMC3507630

[b17] Kowalczyk A. P., Tulloh R. H. & McKeown-Longo P. J. Polarized fibronectin secretion and localized matrix assembly sites correlate with subendothelial matrix formation. Blood 75, 2335–2342 (1990). 2190640

[b18] Kano Y., Katoh K., Masuda M. & Fujiwara K. Macromolecular composition of stress fiber-plasma membrane attachment sites in endothelial cells *in situ*. Circ. Res. 79, 1000–1006 (1996). 8888692 10.1161/01.res.79.5.1000

[b19] Baldus S. . Endothelial transcytosis of myeloperoxidase confers specificity to vascular ECM proteins as targets of tyrosine nitration. J. Clin. Invest. 108, 1759–1770 (2001). 11748259 10.1172/JCI12617 PMC209464

[b20] Rodriguez-Boulan E., Kreitzer G. & Müsch A. Organization of vesicular trafficking in epithelia. Nat. Rev. Mol. Cell Biol. 6, 233–247 (2005). 15738988 10.1038/nrm1593

[b21] De Matteis M. A. & Luini A. Exiting the Golgi complex. Nat. Rev. Mol. Cell Biol. 9, 273–284 (2008). 18354421 10.1038/nrm2378

[b22] Rodriguez-Boulan E. & Macara I. G. Organization and execution of the epithelial polarity programme. Nat. Rev. Mol. Cell Biol. 15, 225–242 (2014). 24651541 10.1038/nrm3775 PMC4211427

[b23] Tiwari A. . Endothelial cell migration on fibronectin is regulated by syntaxin 6-mediated alpha5beta1 integrin recycling. J. Biol. Chem. 286, 36749–36761 (2011). 21880737 10.1074/jbc.M111.260828 PMC3196105

[b24] Riggs K. A. . Regulation of integrin endocytic recycling and chemotactic cell migration by syntaxin 6 and VAMP3 interaction. J. Cell Sci. 125, 3827–3839 (2012). 22573826 10.1242/jcs.102566 PMC3462080

[b25] Reverter M. . Cholesterol regulates Syntaxin 6 trafficking at trans-Golgi network endosomal boundaries. Cell Rep. 7, 883–897 (2014). 24746815 10.1016/j.celrep.2014.03.043

[b26] Shafaq-Zadah M. . Persistent cell migration and adhesion rely on retrograde transport of β1 integrin. Nat. Cell Biol. 18, 54–64 (2016). 26641717 10.1038/ncb3287

[b27] Serra-Pages C., Medley Q. G., Tang M., Hart A. & Streuli M. Liprins, a family of LAR transmembrane protein-tyrosine phosphatase-interacting proteins. J. Biol. Chem. 273, 15611–15620 (1998). 9624153 10.1074/jbc.273.25.15611

[b28] Serra-Pages C. . The LAR transmembrane protein tyrosine phosphatase and a coiled-coil LAR-interacting protein co-localize at focal adhesions. EMBO J. 14, 2827–2838 (1995). 7796809 10.1002/j.1460-2075.1995.tb07282.x PMC398401

[b29] Südhof T. C. The presynaptic active zone. Neuron 75, 11–25 (2012). 22794257 10.1016/j.neuron.2012.06.012 PMC3743085

[b30] Astigarraga S., Hofmeyer K., Farajian R. & Treisman J. E. Three *Drosophila* liprins interact to control synapse formation. J. Neurosci. 30, 15358–15368 (2010). 21084592 10.1523/JNEUROSCI.1862-10.2010 PMC2999520

[b31] Ziv N. E. & Garner C. C. Cellular and molecular mechanisms of presynaptic assembly. Nat. Rev. Neurosci. 5, 385–399 (2004). 15100721 10.1038/nrn1370

[b32] Haucke V., Neher E. & Sigrist S. J. Protein scaffolds in the coupling of synaptic exocytosis and endocytosis. Nat. Rev. Neurosci. 12, 127–138 (2011). 21304549 10.1038/nrn2948

[b33] van der Vaart B. . CFEOM1-associated kinesin KIF21A is a cortical microtubule growth inhibitor. Dev. Cell 27, 145–160 (2013). 24120883 10.1016/j.devcel.2013.09.010

[b34] Clark K. . A specific alpha5beta1-integrin conformation promotes directional integrin translocation and fibronectin matrix formation. J. Cell Sci. 118, 291–300 (2005). 15615773 10.1242/jcs.01623 PMC3329624

[b35] Su Y. . Relating conformation to function in integrin α5β1. Proc. Natl Acad. Sci. USA 113, E3872–E3881 (2016). 27317747 10.1073/pnas.1605074113 PMC4941492

[b36] Byron A. . Anti-integrin monoclonal antibodies. J. Cell Sci. 122, 4009–4011 (2009). 19910492 10.1242/jcs.056770 PMC3329622

[b37] Montanez E. . Kindlin-2 controls bidirectional signaling of integrins. Genes Dev. 22, 1325–1330 (2008). 18483218 10.1101/gad.469408 PMC2377186

[b38] Roberts M., Barry S., Woods A., van der Sluijs P. & Norman J. PDGF-regulated rab4-dependent recycling of alphavbeta3 integrin from early endosomes is necessary for cell adhesion and spreading. Curr. Biol. 11, 1392–1402 (2001). 11566097 10.1016/s0960-9822(01)00442-0

[b39] Arjonen A., Alanko J., Veltel S. & Ivaska J. Distinct recycling of active and inactive β1 integrins. Traffic 13, 610–625 (2012). 22222055 10.1111/j.1600-0854.2012.01327.x PMC3531618

[b40] Wood S. A., Park J. E. & Brown W. J. Brefeldin A causes a microtubule-mediated fusion of the trans-Golgi network and early endosomes. Cell 67, 591–600 (1991). 1657400 10.1016/0092-8674(91)90533-5

[b41] Waguri S. . Visualization of TGN to endosome trafficking through fluorescently labeled MPR and AP-1 in living cells. Mol. Biol. Cell 14, 142–155 (2003). 12529433 10.1091/mbc.E02-06-0338 PMC140234

[b42] Dugina V., Fontao L., Chaponnier C., Vasiliev J. & Gabbiani G. Focal adhesion features during myofibroblastic differentiation are controlled by intracellular and extracellular factors. J. Cell Sci. 114, 3285–3296 (2001). 11591817 10.1242/jcs.114.18.3285

[b43] Tzima E., del Pozo M. A., Shattil S. J., Chien S. & Schwartz M. A. Activation of integrins in endothelial cells by fluid shear stress mediates Rho-dependent cytoskeletal alignment. EMBO J. 20, 4639–4647 (2001). 11532928 10.1093/emboj/20.17.4639 PMC125600

[b44] Pellinen T. . Small GTPase Rab21 regulates cell adhesion and controls endosomal traffic of beta1-integrins. J. Cell Biol. 173, 767–780 (2006). 16754960 10.1083/jcb.200509019 PMC2063892

[b45] Sandri C. . The R-Ras/RIN2/Rab5 complex controls endothelial cell adhesion and morphogenesis via active integrin endocytosis and Rac signaling. Cell Res. 22, 1479–1501 (2012). 22825554 10.1038/cr.2012.110 PMC3463263

[b46] Simpson J. C. . A role for the small GTPase Rab21 in the early endocytic pathway. J. Cell Sci. 117, 6297–6311 (2004). 15561770 10.1242/jcs.01560

[b47] Gravotta D. . Th*e* clathrin adaptor AP-1A mediates basolateral polarity. Dev. Cell 22, 811–823 (2012). 22516199 10.1016/j.devcel.2012.02.004 PMC3690600

[b48] Welz T., Wellbourne-Wood J. & Kerkhoff E. Orchestration of cell surface proteins by Rab11. Trends Cell Biol. 24, 407–415 (2014). 24675420 10.1016/j.tcb.2014.02.004

[b49] Lapierre L. A. . Rab11b resides in a vesicular compartment distinct from Rab11a in parietal cells and other epithelial cells. Exp. Cell Res. 290, 322–331 (2003). 14567990 10.1016/s0014-4827(03)00340-9

[b50] Thuenauer R. . Four-dimensional live imaging of apical biosynthetic trafficking reveals a post-Golgi sorting role of apical endosomal intermediates. Proc. Natl Acad. Sci. USA 111, 4127–4132 (2014). 24591614 10.1073/pnas.1304168111 PMC3964106

[b51] Blum M., De Robertis E. M., Wallingford J. B. & Niehrs C. Morpholinos: antisense and sensibility. Dev. Cell 35, 145–149 (2015). 26506304 10.1016/j.devcel.2015.09.017

[b52] Hoege C. & Hyman A. A. Principles of PAR polarity in *Caenorhabditis elegans* embryos. Nat. Rev. Mol. Cell Biol. 14, 315–322 (2013). 23594951 10.1038/nrm3558

[b53] Tam P. P. & Loebel D. A. Gene function in mouse embryogenesis: get set for gastrulation. Nat. Rev. Genet. 8, 368–381 (2007). 17387317 10.1038/nrg2084

[b54] Hynes R. O. Integrins: bidirectional, allosteric signaling machines. Cell 110, 673–687 (2002). 12297042 10.1016/s0092-8674(02)00971-6

[b55] Lee J. L. & Streuli C. H. Integrins and epithelial cell polarity. J. Cell Sci. 127, 3217–3225 (2014). 24994933 10.1242/jcs.146142 PMC4117227

[b56] Palamidessi A. . The GTPase-activating protein RN-tre controls focal adhesion turnover and cell migration. Curr. Biol. 23, 2355–2364 (2013). 24239119 10.1016/j.cub.2013.09.060

[b57] Parsons J. T., Horwitz A. R. & Schwartz M. A. Cell adhesion: integrating cytoskeletal dynamics and cellular tension. Nat. Rev. Mol. Cell Biol. 11, 633–643 (2010). 20729930 10.1038/nrm2957 PMC2992881

[b58] Trinh L. A. & Stainier D. Y. Fibronectin regulates epithelial organization during myocardial migration in zebrafish. Dev. Cell 6, 371–382 (2004). 15030760 10.1016/s1534-5807(04)00063-2

[b59] Qiao L. . Snail modulates the assembly of fibronectin via α5 integrin for myocardial migration in zebrafish embryos. Sci. Rep. 4, 4470 (2014). 24667151 10.1038/srep04470 PMC3966048

[b60] Jamora C. . Regulation of Golgi structure through heterotrimeric G proteins. Cell 91, 617–626 (1997). 9393855 10.1016/s0092-8674(00)80449-3

[b61] Wierzbicka-Patynowski I., Mao Y. & Schwarzbauer J. E. Analysis of Fibronectin Matrix Assembly. Current Protocols in Cell Biology John Wiley and Sons, Inc. (2004). 10.1002/0471143030.cb1012s25 18228438

[b62] Workman P. . Guidelines for the welfare and use of animals in cancer research. Br. J. Cancer 102, 1555–1577 (2010). 20502460 10.1038/sj.bjc.6605642 PMC2883160

