# Supplementary material for: PPFIA1 drives active α5β1 integrin recycling and controls fibronectin fibrillogenesis and vascular morphogenesis
Source: Nat Commun. 2016 Nov 23;7:13546. doi: 10.1038/ncomms13546 (PMC5122980; doi:10.1038/ncomms13546)
Supplement: Supplementary Information — Supplementary Figures 1 - 14 and Supplementary Tables 1 - 3 [file ncomms13546-s1.pdf]

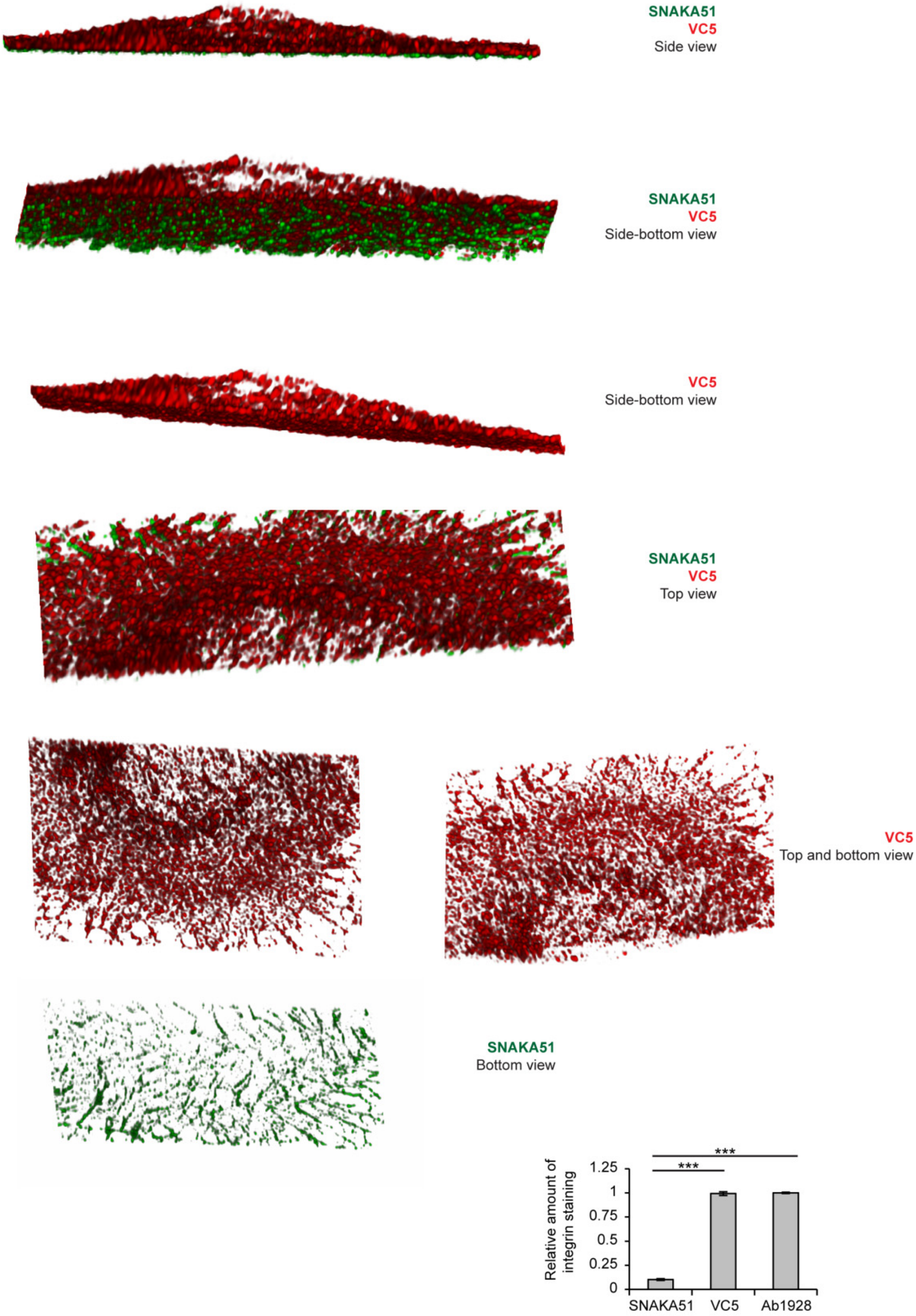

Supplementary Figure 1. 3D reconstruction, distribution, and amount quantification of active and total α5 integrin in ECs. Confocal xy sectioning microscopy analysis and 3D reconstruction of anti-active α5 integrin cell surface localization following 20 minutes of incubation with SNAKA51 mAb (green) and VC5 mAb (red) on living ECs. Side, top, and bottom views as well as quantitative analysis of apico-basal mean fluorescence intensity ratio revealed that while SNAKA51^{+} active α5 integrin localizes on the basolateral surface of ECs, VC5^{+} total α5 integrin is randomly distributed all around the cell surface. Quantitative analysis of apico-basal mean fluorescence intensity ratio of total α5β1 integrin, as recognized by rabbit polyclonal Ab1928 antibody, was performed as further control of VC5 staining. ***P < 0.001, Student's t-test.

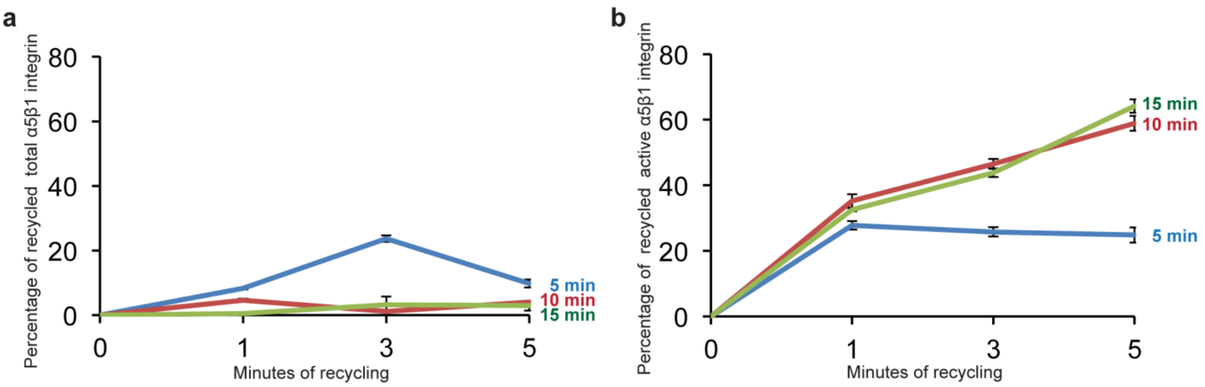

Supplementary Figure 2. In ECs total/inactive, but not active α5β1 integrin recycles mainly from early endosomal compartments. Time-course analysis of the relative amounts of total/inactive (**a**) or active (**b**) α5β1 integrins, as respectively recognized by VC5 and SNAKA51 mAbs, which recycle after being left to be endocytosed for different time points. Total/inactive (**a**), but not active (**b**) α5β1 integrins are only and rapidly recycled to the EC surface within 5 minutes after endocytosis. On the contrary, active (**b**) α5β1 integrins are recycled to the cell surface also at later time points (10 and 15 min). Values are mean ± s.e.m., n = 3 technical replicates. One out of three independent experiments is shown.

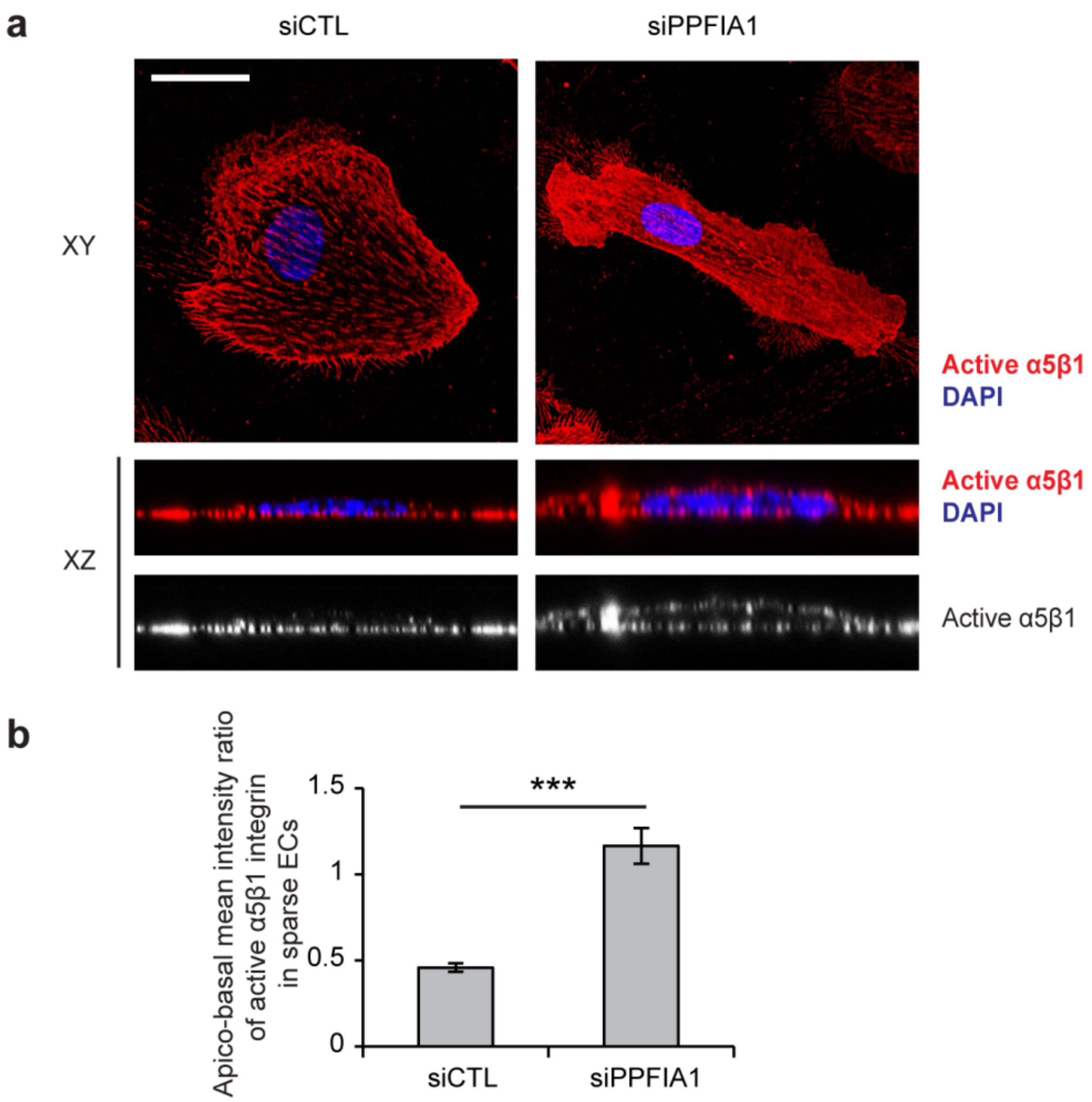

Supplementary Figure 3. PPFIA1 drives basolateral localization of active α5β1 integrin in sparse cells. (**a**) Confocal xy and xz sectioning microscopy analysis of anti-active α5β1 integrin cell surface localization (red) following 20 minutes of incubation with SNAKA51 mAb on living sparse ECs. (**b**) Quantitative analysis of apico-basal mean intensity ratio of SNAKA51^{+} active α5β1 integrin. SNAKA51^{+} active α5β1 integrin localizes on the basolateral surface of sparse siCTL, but not siPPFIA1 ECs, in which it randomly redistributes all around the cell surface. Data are mean ± s.e.m., n = 20 cells per condition pooled from 2 independent experiments. Scale bar, 20 µm (**a**) ***P < 0.001, Student's t-test.

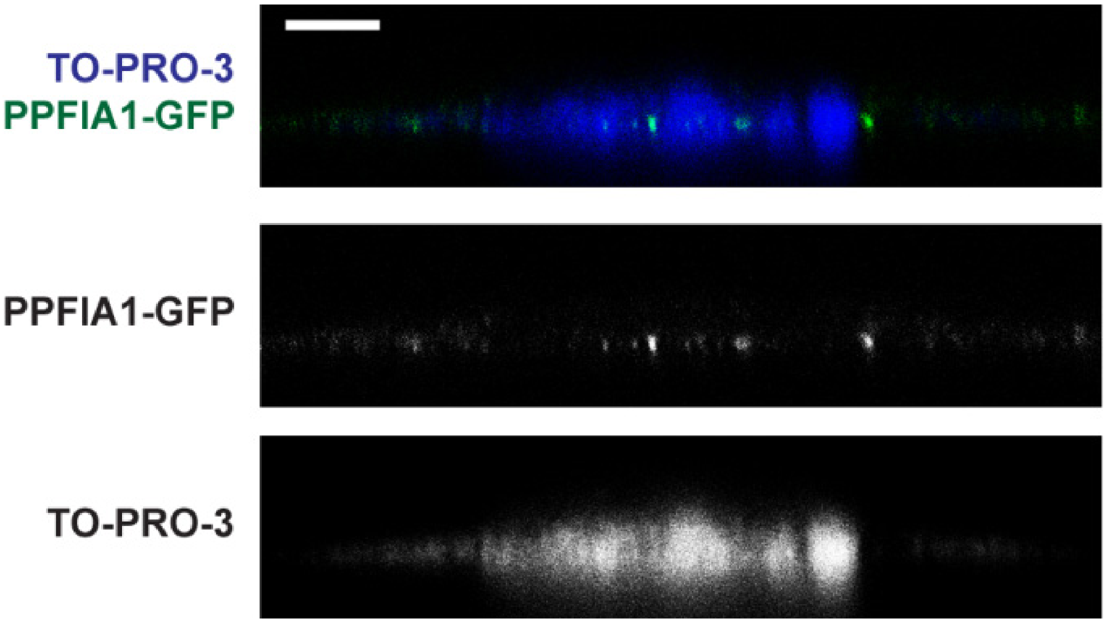

Supplementary Figure 4. PPFIA1 localizes on the basolateral surface of ECs. Confocal xz sectioning microscopy analysis of PPFIA1-GFP localization (green) in confluent ECs that were co-stained with the nuclear marker TO-PRO-3. Scale bar, 5µm

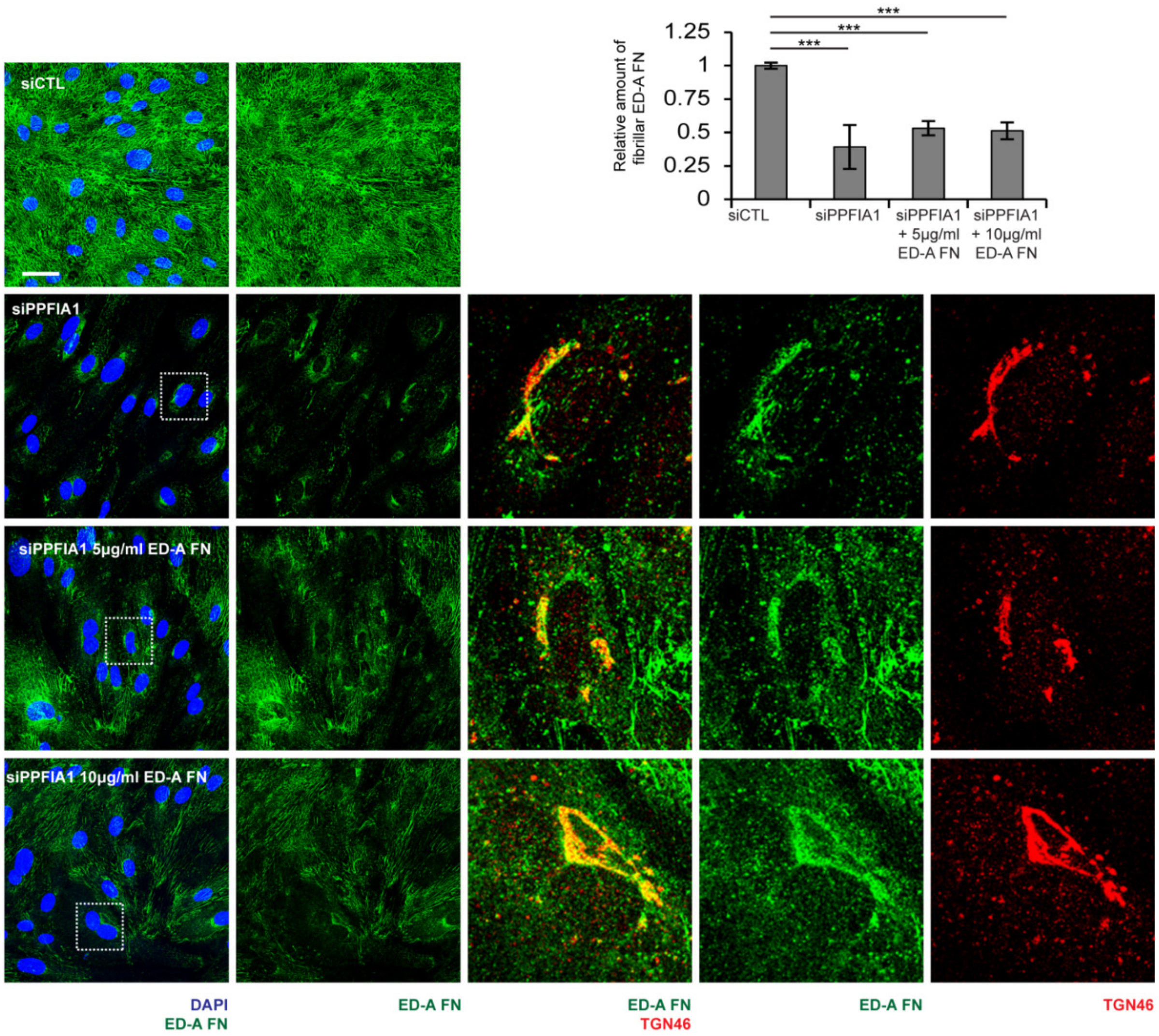

Supplementary Figure 5. Exogenous ED-A FN doesn't rescue basolateral fibrillar FN network in siPPFIA1 ECs. Confocal microscopy analysis of IST-9 mAb-labeled ED-A FN (green) in confluent ECs. ED-A FN polymerizes into a fibrillar network in siCTL, but not in siPPFIA1 ECs in which it accumulates in the TGN46+ (red) TGN cisternae. Exogenously added ED-A FN (5 µg/ml or 10 µg/ml) does not restore basolateral ED-A FN polymerization in siPPFIA1 ECs. The relative amount of fibrillar ED-A FN area was calculated in siCTL, siPPFIA1 and siPPFIA1 + 5 or 10 µg/ml exogenous ED-A FN ECs. Data are mean ± s.e.m., n = 20 cells per condition pooled from 2 independent experiments. Scale bar, 50 µm. ***P < 0.001, Student's t-test.

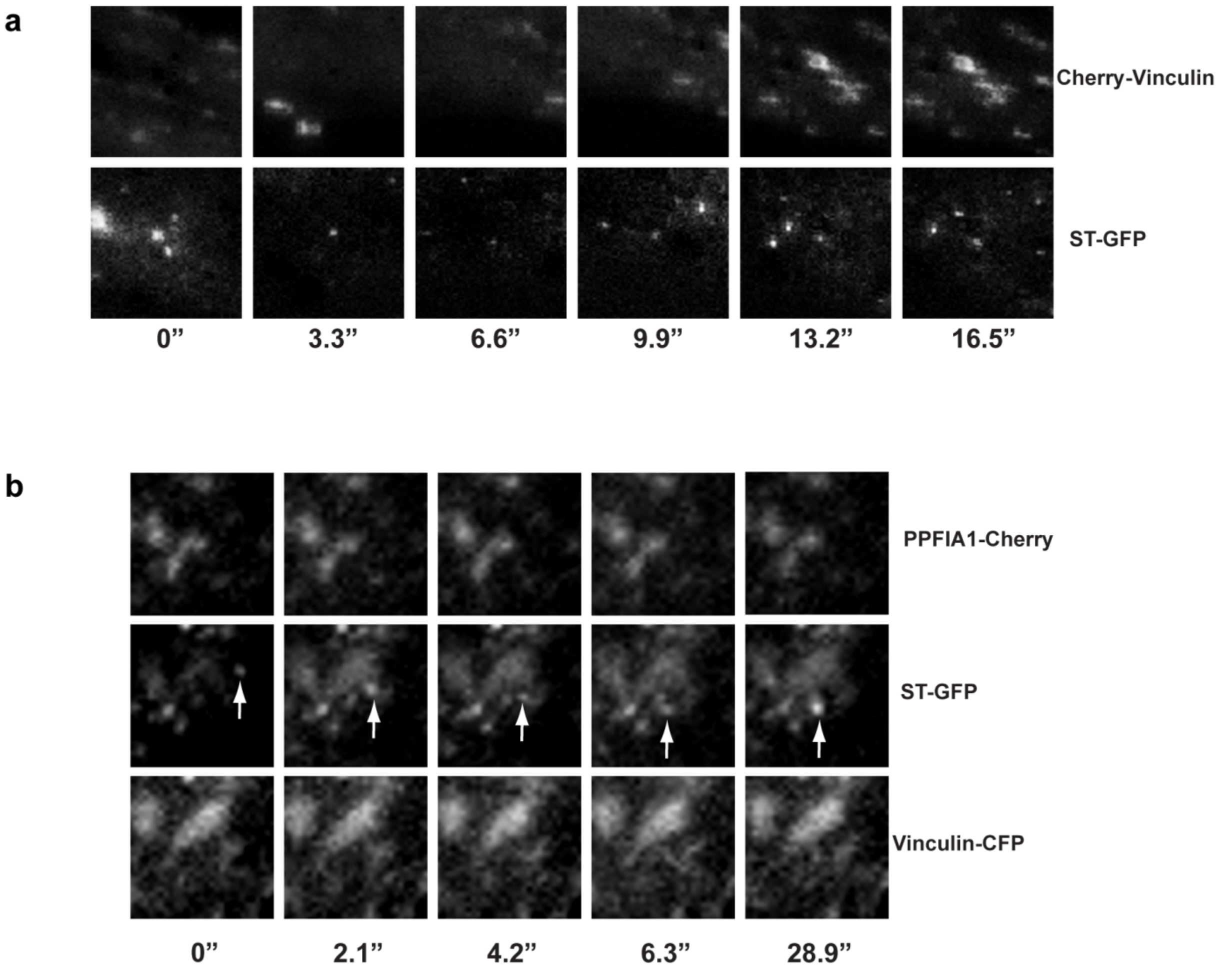

Supplementary Figure 6. Post-Golgi carrier vesicles target endothelial ECM adhesions.
(**a**, **b**) Single channel photograms of the magnifications of the snapshots from live time-lapse total internal reflection fluorescence (TIRF) microscopy shown in Figure 5.

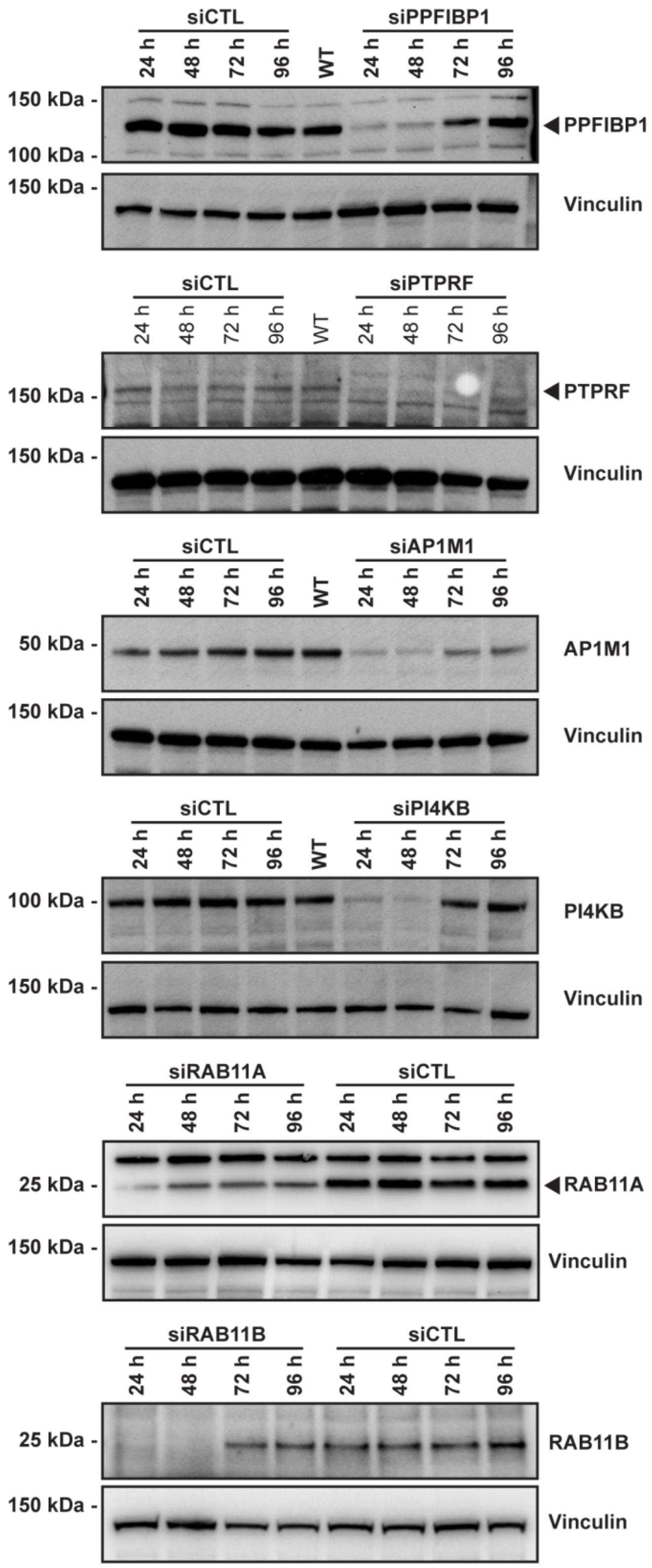

Supplementary Figure 7. Effective siRNA-mediated gene silencing of PPFIBP1, PTPRF, AP1M, PI4KB, RAB11A, and RAB11B proteins in ECs. Western blot analysis of lysates of ECs, control (siCTL), or PPFIBP1 (siPPFIBP1), or PTPRF (siPTPRF), or AP1M1 (siAP1M1), or PI4KB (siPI4KB), or RAB11A (siRAb11A), or RAB11B (siRAB11B) silenced. ECs were lysed 24, 48, 72 and 96 hours after the second siRNA oligofection and proteins were separated by SDS-PAGE and probed for the corresponding Abs. Where more bands are present in Western Blots the arrowhead indicates the one corresponding to the specific protein.

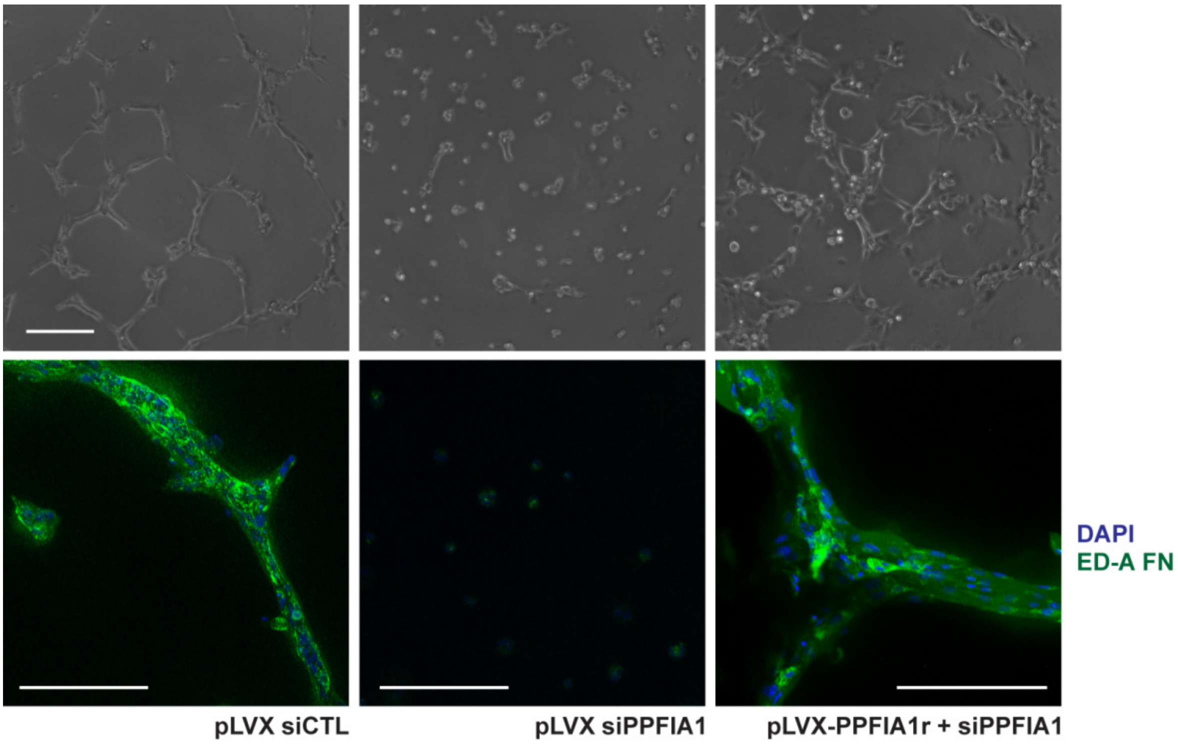

Supplementary Figure 8. PPFIA1 silencing affects vascular morphogenesis in cultured ECs. Representative pictures of vascular networks formed by pLVX siCTL, pLVX siPPFIA1, and pLVX- PPFIA1r + siPPFIA1 ECs plated on growth factor-reduced Matrigel matrix. PLVX siCTL but not pLVX siPPFIA1 ECs form capillary networks that are covered by a dense meshwork of polymerized cellular ED-A fibronectin. PLVX-mediated PPFIA1r overexpression restores capillary network formation and ED-A FN polymerization by siPPFIA1 ECs. Scale bar, 100 µm.

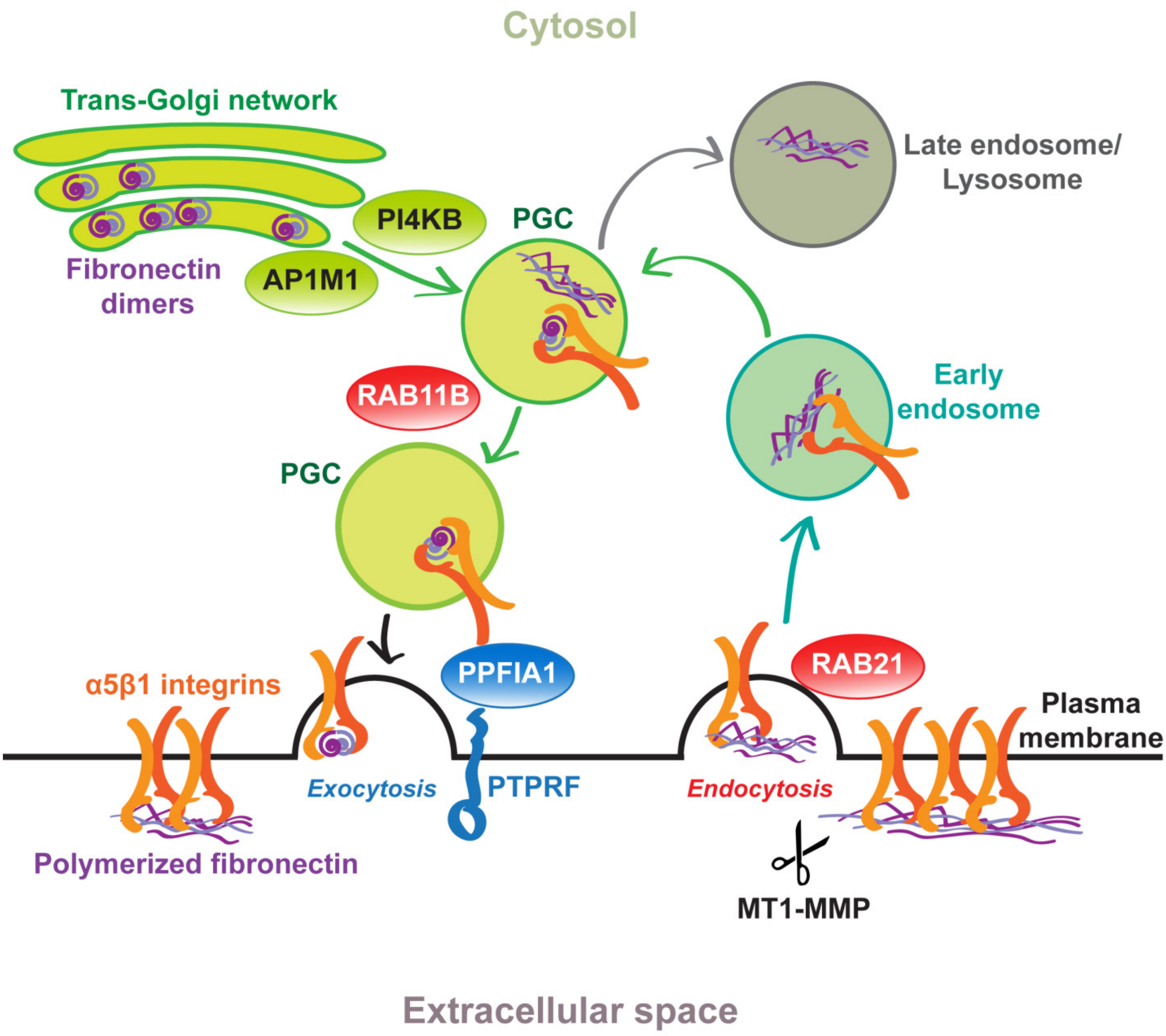

Supplementary Figure 9. A TGN-hinged signaling pathway couples active α5β1 integrin traffic and fibronectin fibril turnover. In ECs, upon MT1-MMP-dependent cleavage of FN fibrils, FN fragment-bound active α5β1 integrins undergo Rab21 GTPase-driven internalization within early endosomes. From this subcellular location, FN fragment-bound active α5β1 integrins traffic to post-Golgi vesicles (PGCs) that bud, in a PI4KB and AP-1A-dependent manner, from the Trans-Golgi network (TGN) cisternae and contain freshly synthesized FN. Within PGCs, active α5β1 integrins may swap old FN fragments for new FN dimers and the former are likely directed towards the late endosomal/lysosomal compartments for degradation. PGCs containing fresh FN-bound active α5β1 integrins are then directed, on a RAB11B-dependent pathway, towards the basolateral side of ECs. Here, the PTPRF/PPFIA1 complex localizes in close proximity of fibrillar adhesions. Similarly to the function that it plays in neuron presynaptic sites and thanks to its ability to bind the β1 cytotail of active α5β1 integrin, PPFIA1 may favor the docking of PGCs containing fresh FN-bound active α5β1 integrins. The ensuing fusion of PGCs with the plasma membrane may favor the targeted local appearance of fresh FN-bound active α5β1 integrins, thus allowing the replacement of old for new FN within fibrils. Finally, a corollary hypothesis may be that, upon endocytosis, active α5β1 integrin-bound non-polymerized FN dimers recycle back to the basolateral EC surface and that this endo-exocytic cycle continues until FN dimers incorporate into fibrils due to polymerization (not depicted).

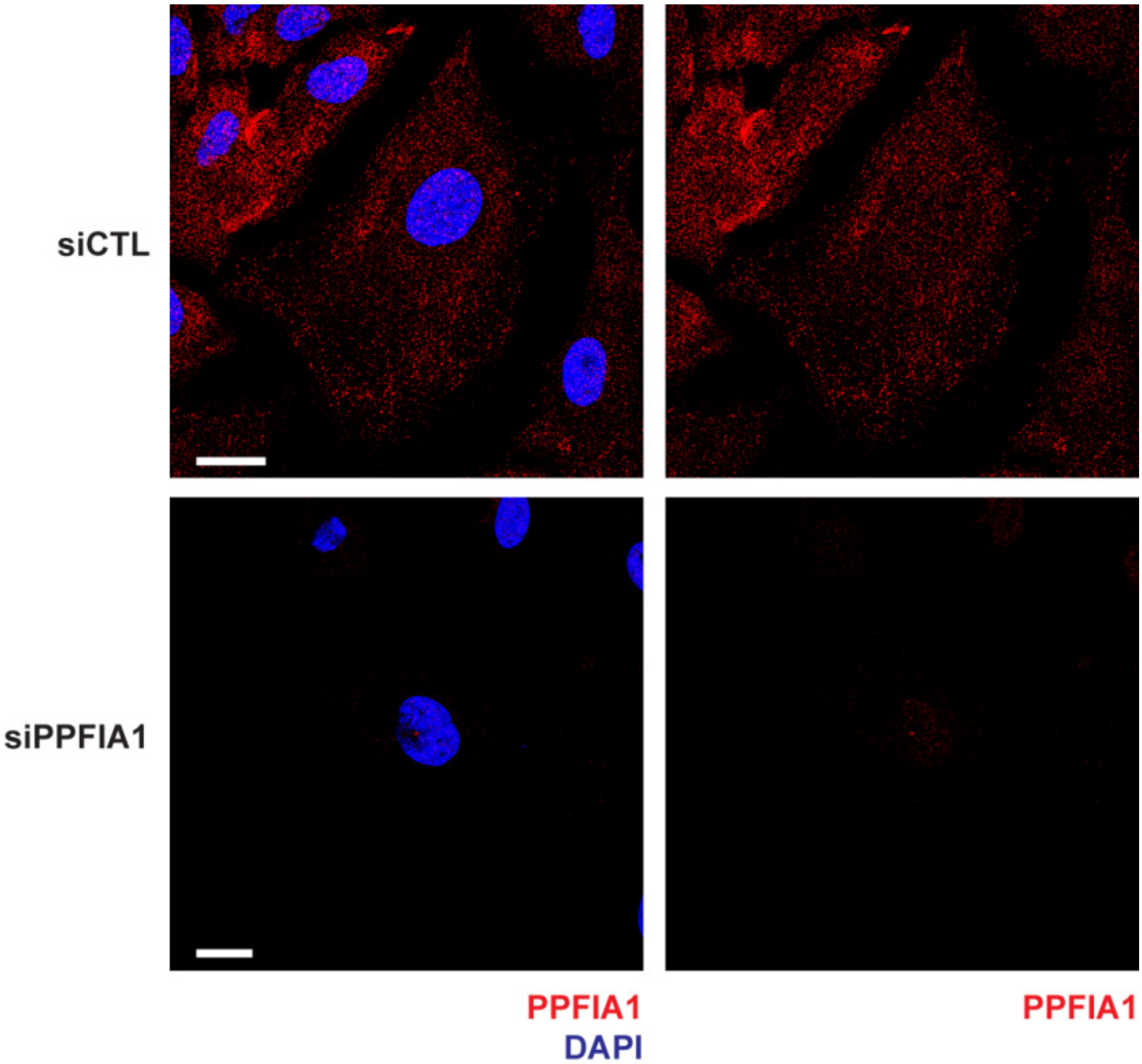

Supplementary Figure 10. Characterization of rabbit polyclonal anti-PPFIA1 antibody. siCTL or siPPFIA1 ECs were fixed and stained with rabbit polyclonal antibody anti-PPFIA1 and DAPI. Confocal fluorescence microscopy analysis demonstrate that specific PPFIA1 staining present in siCTL ECs is totally absent in siPPFIA1 ECs. Scale bar, 20µm.

Figure 1, panel d

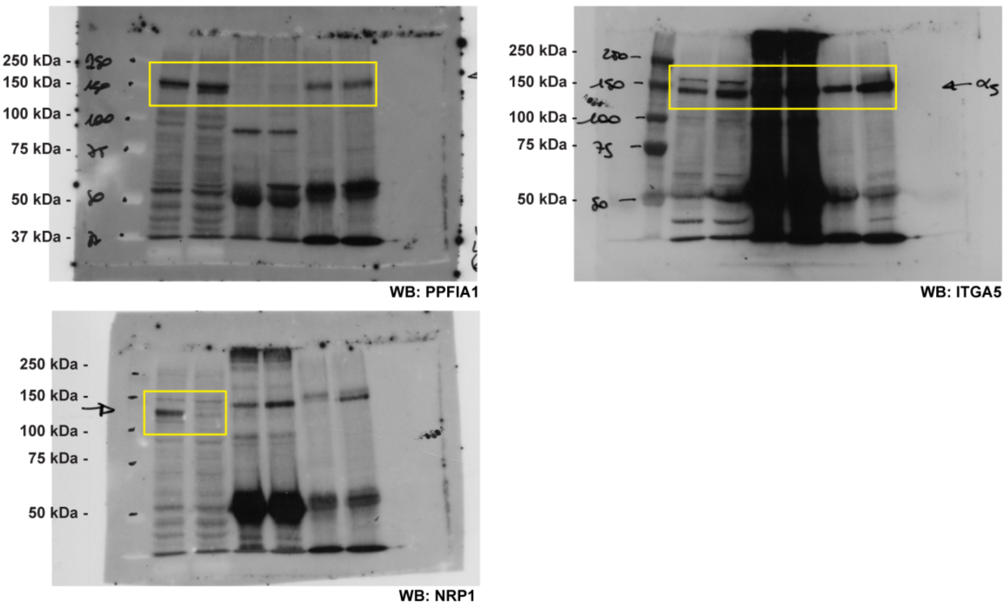

Figure 1, panel e

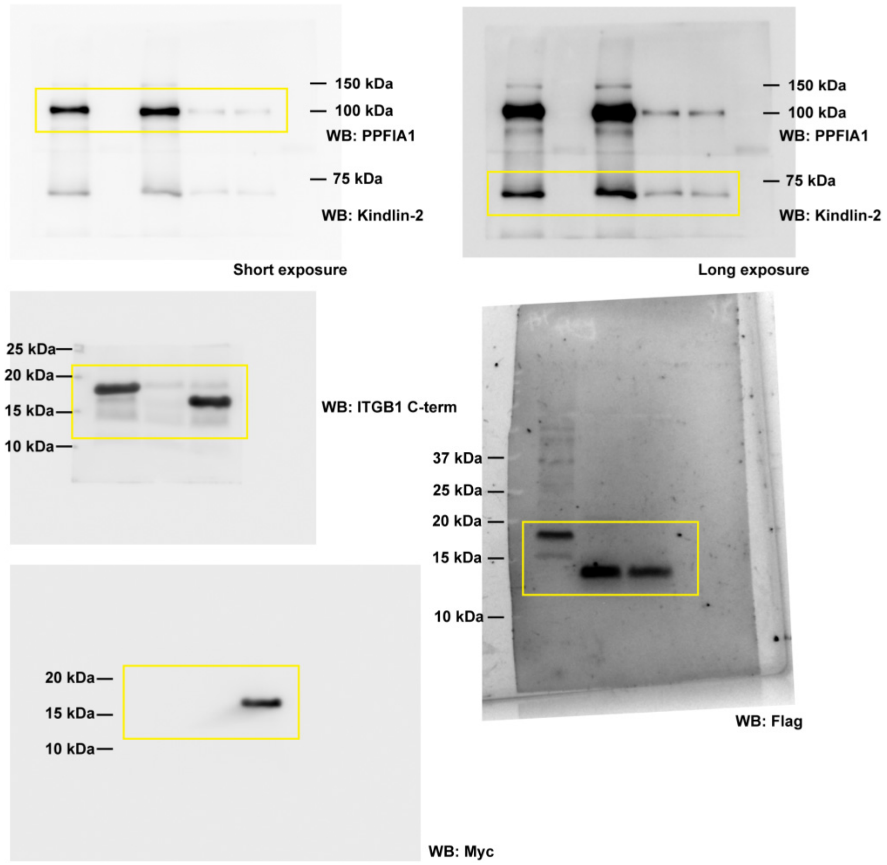

Supplementary Figure 11. Full images of blots that were cropped in main Figure 1. Yellow squares indicate the cropped images.

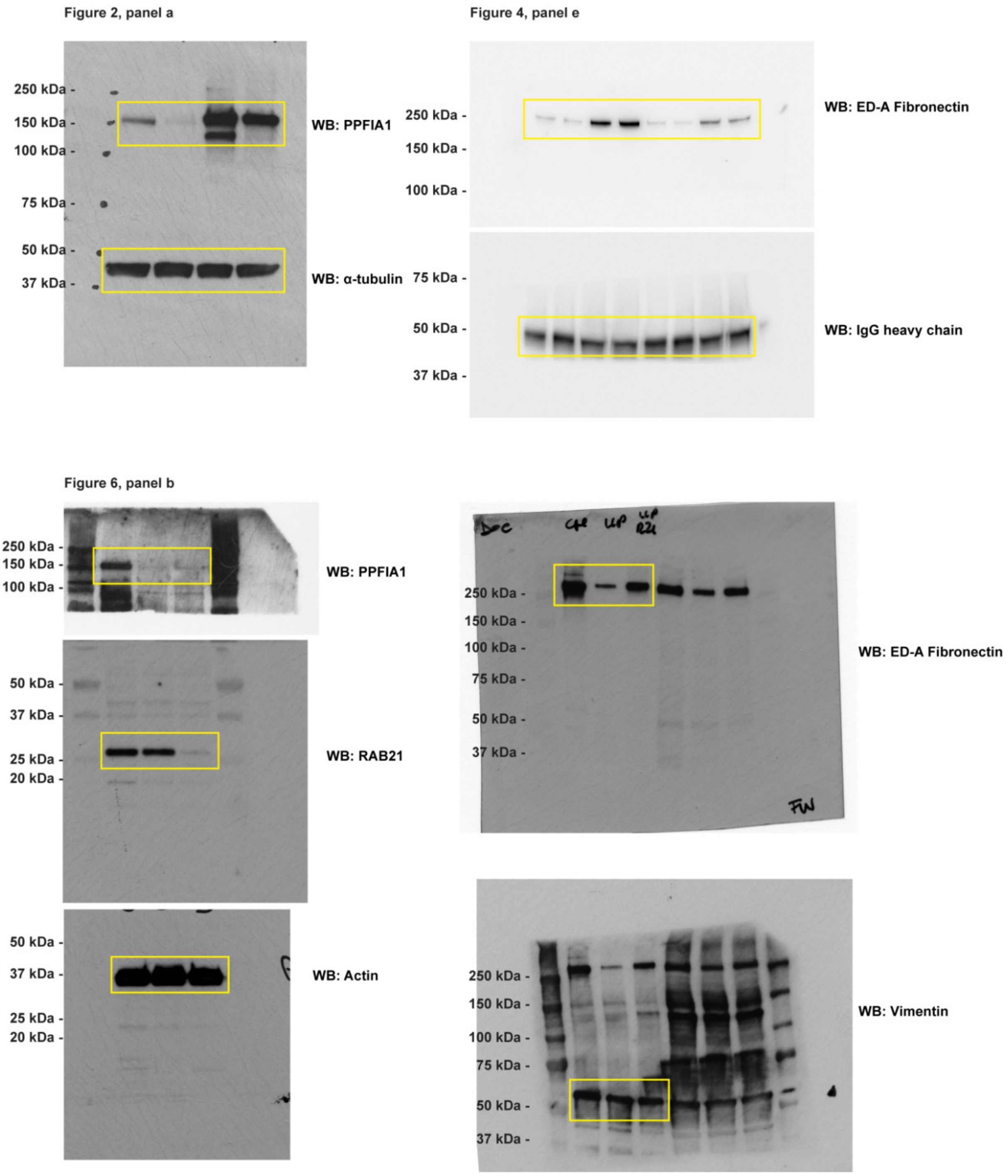

Supplementary Figure 12. Full images of blots that were cropped in main Figures 2, 4, and 6. Yellow squares indicate the cropped images.

Figure 8, panel e

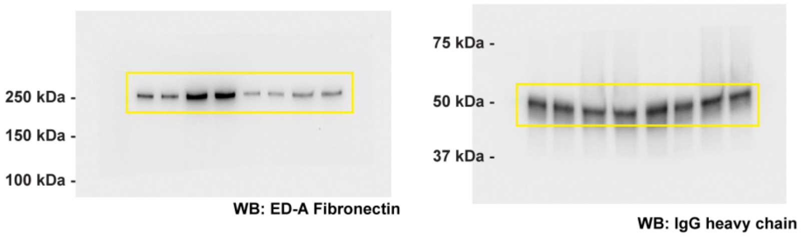

Figure 9, panel a

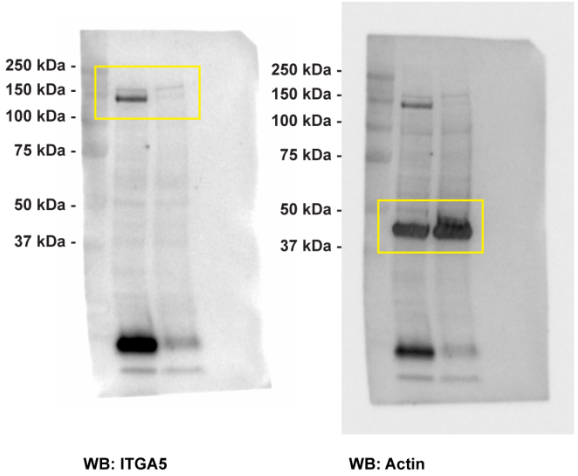

Figure 9, panel c

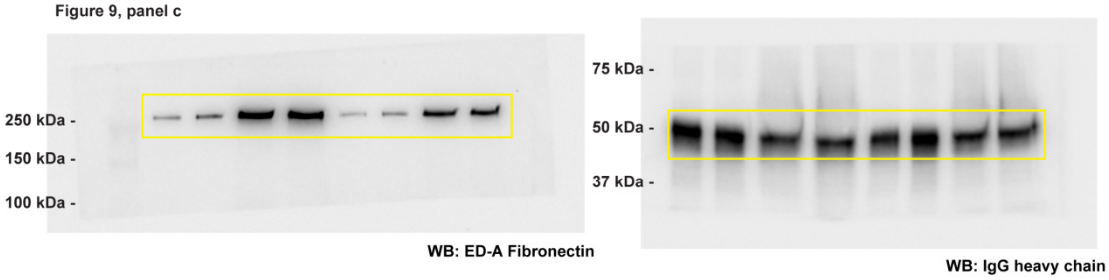

Figure 10, panel b

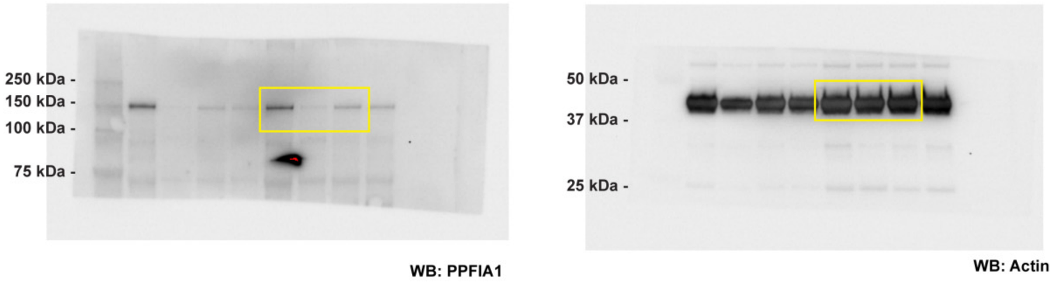

Supplementary Figure 13. Full images of blots that were cropped in main Figures 8, 9, and 10. Yellow squares indicate the cropped images.

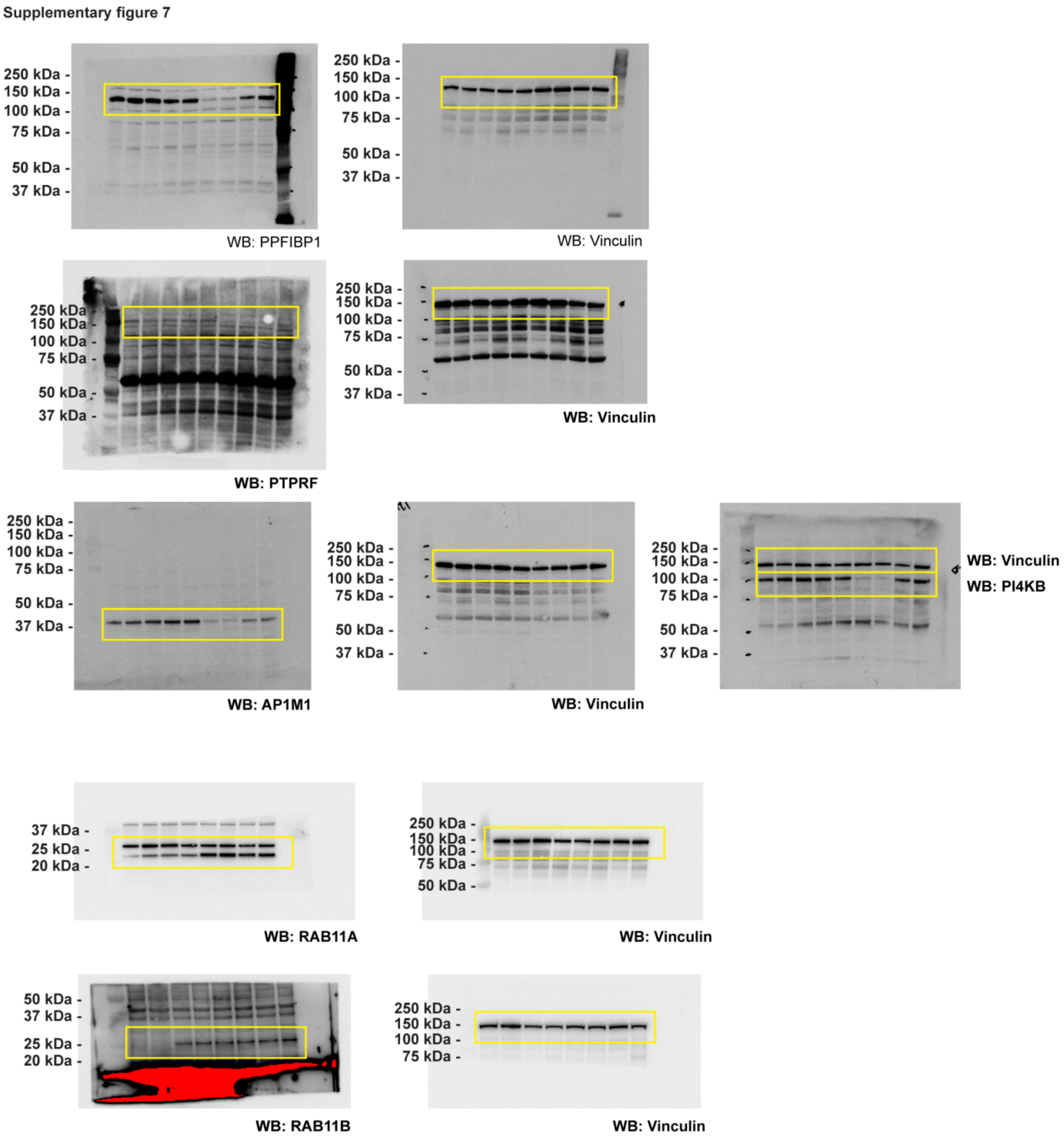

Supplementary Figure 14. Full images of blots that were cropped in Supplementary Figure 7. Yellow squares indicate the cropped images.

	MO CTL	MO *ppfia1*	MO *ppfia1* + mRNA *PPFIA1*
**Injected eggs**	226	467	585
**Total embryos after 72hpi**	154	252	307
**Normal embryos**	148	166	261
**Altered embryos**	6	84	47

Supplementary Table 1. Absolute number of MO-CTL, MO-*ppfia1* and MO-*ppfia1*+*PPFIA1* embryos having normal or altered phenotype.

	MO CTL	MO *ppfia1*	MO *ppfia1* + mRNA *PPFIA1*
**Normal embryos**	96	66	84
**Altered embryos**	3,8	33,6	15,3

Supplementary Table 2. Relative Percentage of MO-CTL, MO-*ppfia1* and MO-*ppfia1*+*PPFIA1* embryos having normal or altered phenotype.

Antigen	Antibody	Provider	WB	IF	IP
**Actin**	NB100-74340	Novus Biologicals	1:5000		
**AP1M1**	12112-1-AP	PTG	1:1000		
**EEA1**	sc-6415	Santa Cruz		1:200	
**Fibronectin**	F3648	Sigma		1:200	
**Fibronectin**	sc-59826 (clone IST9)	Santa Cruz	1:1000	1:100	
**Flag**	A8592 (clone M2)	Sigma	1:1000		
**ITGA5**	AB1949	Millipore	1:1000		
**ITGA5**	SNAKA51	Martin J. Humphries' lab		1:100	1µg/ml
**ITGA5**	555651 (clone VC5)	BD Biosciences			1µg/ml
**ITGB1**	AB1952	Millipore	1:2000		
**Kindlin-2**	Mab2617	Millipore	1:1000		
**LAMP1**	555798	BD Biosciences		1:50	
**Myc**	05-419 (clone 9E10)	Millipore	1:1000		
**NRP1**	sc-7239	Santa Cruz	1:1000		
**PI4KB**	NBP2-12814	Novus Biologicals	1:1000		
**PPFIA1**	14175-1-AP	PTG	1:1000	1:50	
**PPFIBP1**	13961-1-AP	PTG	1:1000		
**PTPRF**	sc-135969	Santa Cruz	1:500		
**Rab11A**	TA324158	Origene	1:500		
**Rab11B**	TA346586	Origene	1:500		
**Rab21**	Rabbit polyclonal	Johanna Ivaska's lab	1:2000		
**TGN46**	AHP1586	ABD Serotec		1:100	
**TGN46**	AHP500	ABD Serotec		1:100	
**Vimentin**	V5255	Sigma	1:1000		
**Vinculin**	V9264	Sigma	1:1000	1:400	
**α-Tubulin**	T5168	Sigma	1:2000		

Supplementary Table 3. Antigen, provider, and dilution used in different experimental settings for all the commercial and non-commercial primary antibodies employed in this study. Western blot (WB), immunofluorescence (IF), and immunoprecipitation (IP).
